# Heat treatment as a universal technical solution for silcrete use? A comparison between silcrete from the Western Cape (South Africa) and the Kalahari (Botswana)

**DOI:** 10.1371/journal.pone.0181586

**Published:** 2017-07-19

**Authors:** Patrick Schmidt, David J. Nash, Sheila Coulson, Matthias B. Göden, Graeme J. Awcock

**Affiliations:** 1 Department of Prehistory and Quaternary Ecology, Eberhard Karls University of Tübingen, Tübingen, Germany; 2 Department of Geosciences, Applied Mineralogy, Eberhard Karls University of Tübingen, Tübingen, Germany; 3 School of Environment and Technology, University of Brighton, Brighton, United Kingdom; 4 School of Geography, Archaeology and Environmental Studies, University of the Witwatersrand, Johannesburg, South Africa; 5 Department of Archaeology, Institute of Archaeology, Conservation and History, University of Oslo, Oslo, Norway; Max Planck Institute for the Science of Human History, GERMANY

## Abstract

Heat treatment was one of the first transformative technologies in the southern African Middle Stone Age (MSA), with many studies in the Cape coastal zone of South Africa identifying it as an essential step in the preparation of silcrete prior to its use in stone tool manufacture. To date, however, no studies have investigated whether heat treatment is necessary for all silcrete types, and how geographically widespread heat treatment was in the subcontinent. The aim of this study is to investigate experimentally whether heat treatment continued further north into the Kalahari Desert of Botswana and northernmost South Africa, the closest area with major silcrete outcrops to the Cape. For this we analyse the thermal transformations of silcrete from both regions, proposing a comprehensive model of the chemical, crystallographic and ‘water’-related processes taking place upon heat treatment. For the first time, we also explore the mobility of minor and trace elements during heat treatment and introduce a previously undescribed mechanism—steam leaching—causing depletion of a limited number of elements. The results of this comparative study reveal the Cape and Kalahari silcrete to respond fundamentally differently to heat treatment. While the former can be significantly improved by heat, the latter is deteriorated in terms of knapping quality. These findings have important implications for our understanding of the role of fire as a technical solution in MSA stone tool knapping, and for the extension of its use in southern Africa. Silcrete heat treatment—at least in the form we understand it today—may have been a strictly regional phenomenon, confined to a narrow zone along the west and south coast of the Cape. On the basis of our findings, silcrete heat treatment should not be added as a new trait on the list of behaviours that characterise the MSA of the southern African subcontinent.

## Introduction

Heat treatment was one of the first transformative technologies employed by our human ancestors in the southern African Middle Stone Age (MSA), with its use argued to shed light on socioeconomic processes, early resource management, and even cognitive and social capacities [1–6]. Its main application was to improve the flaking qualities of silcrete, a raw material used in tool manufacture throughout the Stone Age in the subcontinent. Controlled experiments on samples from the Cape coastal zone of South Africa indicate that silcrete in this area responds to heat treatment with significant improvement in workability, primarily due to the loss of chemically bound water during heating, and the formation of new Si-O-Si silica bonds [7, 8]. So far, six Middle and Late Stone Age (LSA) sites in South Africa have yielded published evidence of silcrete heat treatment—Pinnacle Point [3]; Diepkloof Rock Shelter [9]; Mertenhof Rock Shelter [10]; Klipdrift Shelter [11]; Blombos Cave [12]; Elands Bay Cave [13]–and publications on several other sites are expected in the near future (Schmidt, personal data). Strikingly, in some of the assemblages of these sites, the vast majority of silcrete was heated prior to use. Because of this abundance of heat-treated silcrete in the archaeological record of the Cape, silcrete has, for some, acquired the status of a material that needs to be heat-treated to be knappable [14] (for pressure flaking [15]). To date, however, it has not been investigated as to whether heat treatment is necessary for all silcrete types, and how geographically widespread heat treatment was in the subcontinent.

In this study we compare experimentally the response to heat treatment of silcretes from the two main silcrete provinces in southern Africa [16]: the Middle Kalahari of Botswana, and the Cape coastal zone of South Africa. The aim of our comparison is to investigate whether the thermal transformations arising from heat treatment, and the parameters they impose to a heat-treating individual, are the same in the two regions. Silcrete is known to have formed by different mechanisms in the Cape and the southern African interior. In the southern and western Cape, silcrete formation was predominantly by pedogenic processes operating upon a range of deeply weathered lithologies associated with ancient palaeosurfaces [17, 18]. In the interior, in contrast, the silicification of Kalahari Group sediments occurred mainly in the vicinity of landscape depressions (i.e. river valleys, lakes and pans) via non-pedogenic processes [16, 19–22]. Silcretes from the two regions also exhibit differing chemistries [23, 24] and petrographic features [16]. Intuitively, therefore, they might be expected to display different thermal transformations in response to heating. If this is the case, the role of heat treatment as a technical solution for the use of silcrete in stone tool manufacture will require review.

## Materials and methods

### Samples and sample preparation

For this study, two samples of silcrete were collected from the Western Cape Province of South Africa and two from North-West District in Botswana. No permissions were required for collecting these rock samples as silcrete is not a rare or precious resource either of both countries. The samples from the Western Cape were chosen because the two selected silcrete outcrops had been used for raw material provisioning at Diepkloof Rock Shelter and Elands Bay Cave, and the silcrete at these sites was frequently heat-treated by MSA and LSA occupants [9, 13, 25]. Both samples were collected in primary position from two weathering profiles near the towns of Hopefield and Redelinghuys, and are typical pedogenic silcretes [17, 18]. The samples from Botswana were collected in primary position at two sites along the Boteti River, close to the village of Samedupi, and are representative of drainage-line silcrete, the most widespread variety of non-pedogenic silcrete found in the Kalahari [26]. Silcrete from the Boteti River is known to have been used as a raw material source by the peoples who occupied MSA sites in the Tsodilo Hills [27, 28], and, to a lesser extent, the MSA site at #Gi [28–31]. Sample numbers and petrographic descriptions (one standard thin-section was cut from each sample) are summarised in Table 1. The aim of our experiment was to compare the thermal transformations in samples from the two regions. This comparison was to be made in terms of ‘water’-related thermal transformations (as assessed by IR-spectroscopy [7]) and, concomitantly, the stability of minor and trace elements in the samples (as assessed by atomic- and mass-spectrometry [27]). Sample preparation therefore needed to follow a protocol to produce IR- and geochemistry-samples that could be compared but were not influenced by within-sample heterogeneity.

**Table 1:** Silcrete sample numbers, origins and petrographic descriptions.

Sample No.	Location	Description
**WK-13-13**	South Africa, West Coast, near the town of Redelinghuys	Very poorly sorted pedogenic silcrete, with grain-supported (GS-) to F-fabric. Clasts: ~70 vol% with average size ~0.92 mm. Cement: microquartz with crystal size of <5 μm.
**WK-15-01**	South Africa, West Coast, near the town of Hopefield	Very poorly sorted pedogenic silcrete, with floating (F-) fabric. Clasts: 30–40 vol% with average size ~0.2 mm. Cement: microquartz with crystal size of ~15 μm.
**BW-14-01**	Botswana, North-West District, Samedupi Drift, Boteti River	Well-sorted drainage-line silcrete with GS-fabric. Clasts: ~80 vol% with average size ~0.25 mm. Cement: epitaxial LS-chalcedony overgrowth on clasts, void filling LF-chalcedony
**BW-14-02**	Botswana, North-West District, Samedupi Drift, Boteti River	Well-sorted drainage-line silcrete with GS-fabric. Clasts: ~80 vol% with average size ~0.25 mm. Cement: epitaxial LS-chalcedony overgrowth on clasts, void filling LF-chalcedony

**Fig 1:**
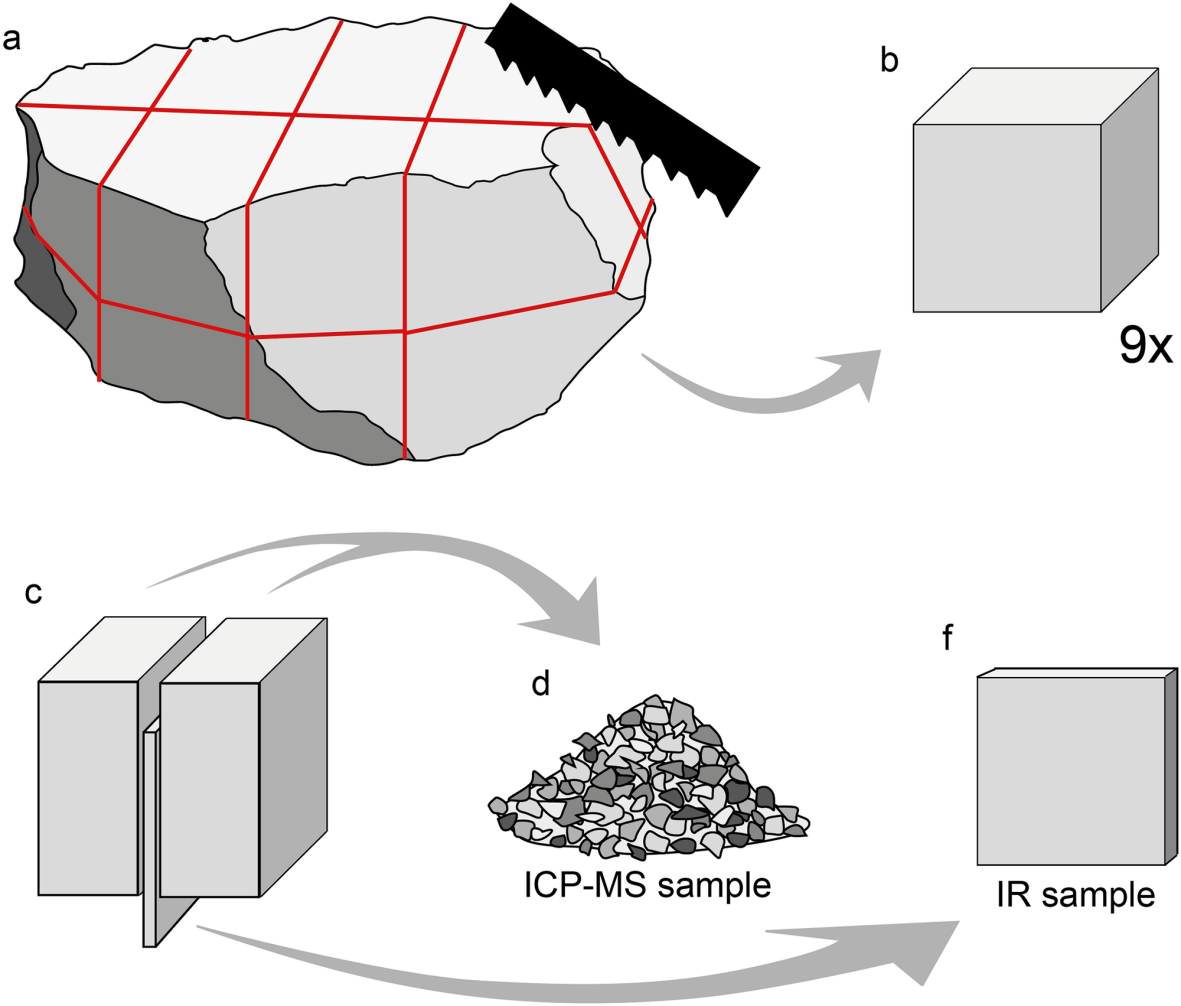
Protocol used for sample preparation. Each ~20 cm diameter sample block (a) was cut into nine ~3 cm cubes (b). Each cube was cut into half-cubes, and a thin slab cut from its centre (c). Both half-cubes from each cube were crushed together and homogenised for geochemical analysis (d), and the slab was kept for infrared spectroscopy and colour change analysis. This protocol allows for direct comparability of geochemical and infrared samples, minimizing the effects of sample heterogeneity.

The protocol for sample preparation is shown in Fig 1. For this, nine cubes with an average edge length of ~3 cm were cut from each of the four original blocks of silcrete (each ~20 cm diameter). The average volume of each cube was ~27 cm^3^ and the mass ~70 g. Each of the cubes was then cut approximately in half, with a thin slab (originally situated across the centre of the cube) removed from one of the half-cubes. Each of these slabs was lapped to a plan-parallel section and diamond polished on both sides. The polished slabs were used for transmission IR-spectroscopy and the analysis of colour change. The remaining two half-cubes were mechanically crushed into a granular mass made of fragments with an average size of ~1 cm (henceforth called a ‘granular’ for simplicity). The granulars obtained from both half-cubes were mixed together, homogenised and sieved to keep only the fraction > 3 mm < 15 mm for geochemical analysis.

The main experiment (see ‘Experimental protocol‘) required that both the polished slab and granulars resulting from each cube were heat-treated together, with samples from different cubes heated to different temperatures. Although heterogeneity of the minor and trace element concentration within a single cube cannot be ruled out, the step of mixing and homogenisation was intended to provide uniform granulars that were comparable to the polished IR-slab from the same cube. We chose a relatively large fragment size for the granulars over finer powders, as the thermal transformations in these fragments should be more comparable with larger samples. Pelto [32] showed that in chalcedony, for example, even smaller particles build up internal steam pressure during heat treatment, creating a closed system comparable to larger samples. The masses of the granulars and thicknesses of the polished slabs are summarised in S1 Table.

Following sample preparation, IR- and geochemistry-samples underwent the experimental protocol detailed in section 2.2. After these experiments, but prior to geochemical analysis, each granular was fine-crushed in an agate mill (to 70% passing <2 mm or better), split using a rotary splitter and then pulverised in an agate mill (to 85% passing 75 μm or better). The surplus of the powders remaining after geochemical analysis was used for X-ray powder diffraction to evaluate the thermal evolution of crystallinity in each sample.

### Experimental protocol

For the experiment, each granular was first separated into two aliquots. One of these was used as a control, and was analysed for minor and trace element concentrations without further treatment; the other was heat-treated to a target temperature and then analysed. Target temperatures were: 110°C, 200°C, 250°C, 300°C, 350°C, 400°C, 450°C, 500°C and 600°C. We chose a heating rate of 4°C/min to avoid unwanted fracturing (see for example [8, 9]) and a 2 hours dwell time at maximum temperature for all ‘water’-related transformations to be complete (based on the results of [33]).

In parallel, we measured all IR-samples (i.e. the 36 polished slabs) before heat treatment. Spectra were acquired in two states: (i) the slabs were dehydrated at 110°C for 48h, for all loosely held H_2_O to be evacuated from the open pore space, and then measured by IR-spectroscopy. This is the ‘dehydrated state’ measurement (Dehy). (ii) We then rehydrated the slabs in deionised H_2_O for 24h at 60°C (1 bar) and re-measured them. This is the ‘rehydrated state’ measurement (Rehy). In addition to providing data on different water species, this two-step measuring protocol evaluates the water-adsorbing porosity of each sample [7, 34]. Following this first set of ‘before-heating’ IR measurements, each IR-sample was heat-treated to a target temperature along with one of the granular aliquots from the corresponding cube. After cooling to room temperature, each heat-treated IR-sample was measured a second time in its Dehy- and Rehy-states. Element, ‘water’ and porosity concentrations measured after heat treatment were then subtracted from the concentrations measured before heat treatment to detect differences, i.e. thermal transformations. This protocol provides data on the stability or loss of minor and trace elements within each cube that can be compared with its porosity- and ‘water’-related transformations.

In parallel, we qualitatively evaluated the roughness of fresh fracture surfaces of two samples heated to different temperatures. For this, we first knapped a control flake from each of samples WK-15-01 and BW-14-02. Both samples were then heat-treated for 2 hours at 300°C with a ramp of 4°C/min (choice of these conditions explained above). A second flake was removed from each sample after this first treatment. The blocks were then heat-treated at 400°C and 500°C (same ramp and dwell time), with a third and fourth flake knapped after each temperature step. We compared and photographed the surface roughness of these flakes in identical raking light conditions to obtain an estimation of the flaking mechanics and, consequently, the knapping quality of each sample.

### Instruments and data treatment

#### Colour change

Following experimental heat treatment, images of each of the 36 polished slabs were analysed to assess the extent of any colour change that may have occurred during heating (see S1 Text for full details of image capture and analysis protocols). To achieve this, each polished slab was first photographed against an X-Rite ColorChecker Passport calibration target background, under a custom-built hemispheric white LED light source. Hemispheric lighting was used to provide near-uniform illumination, standardise image acquisition and better support calibration. Images were acquired in RAW image format, using a Canon 400D SLR camera fitted with a 60 mm macro lens, with a standardised exposure time of 1/3 second at f8 aperture.

RAW images were processed within the ImageJ image processing environment. Images were first normalised to absolute reflectance values, using a customised processing workflow based on Troscianko and Stevens [35]. This involved the development of custom scripts that integrated compensation for lighting non-uniformity into the workflow, through the use of a Flat-Field-correction (FFC) strategy supported by images of a uniform white-reference target. Linearity of the processed output images was checked using six target grey patches of known reflectance (viz 89.1%, 58.9%, 36.3%, 20.0%, 8.9% and 3.2%) from the X-Rite ColorChecker Passport calibration target. For Red, Green and Blue (R, G, B) colour channels, the linearity of the processed outputs was improved by the use of FFC, with R^2^ values of the linear fit raised to 0.9998, 0.9994 and 0.9985, respectively.

The processed RAW images were then examined for red-dominance. Wherever possible, a common 396 x 405 pixel region-of-interest (ROI) within each processed image was used as the target of analysis. R, G, B histograms of the ROI for each sample were scrutinised using a semi-automated scripted process, and the dominant modal reflectance values recorded. The dominant mode was selected to eliminate bias in the mean value caused by large inclusions present in some images. The results presented in section ‘Colour change upon heat treatment’ depict the saturated red reflectance (i.e. the dominant modal R value minus the minimum R, G, B value within the ROI) as a percentage of the saturated colour magnitude (the vector magnitude of the saturated R and G reflectance values, since B reflectance was always the minimum R, G, B dominant modal value) against the sample treatment temperature. This value was selected to eliminate the influence of scene intensity.

#### X-ray powder diffraction (XRD)

Powder diffractograms between 5 and 70°2*ϴ* were recorded using a PANalytical X’Pert Pro diffractometer (using the Kα line of a Cu anode, no incident beam monochromator). The sample holder was in constant rotation during the analysis. We did not calculate crystal size with the Scherrer equation [36] because it does not distinguish between X-ray line broadening due to crystal-size and -strain. Instead, we calculated internal strain using the Williamsons-Hall method [37], which is based on the principle that internal strain causes different line broadening of diffraction peaks at different diffraction angles [38]. Williamsons-Hall plots also provide an estimation of the mean coherent scattering domain, or average ‘grain size’. However, it should be kept in mind that, in silcrete, this value may give a false impression of an actual measurement of crystal size. The measurements were obtained by bulk rock analyses that average out quartz clasts and matrix grains. We, therefore, plotted coherent scattering domain size values after heating to different temperatures only to understand their relative thermal evolution. For the calculations we used the peaks at (in 2*ϴ*): 20.7° $\left(10\bar{1}0\right)$; 26.6° $\left(10\bar{1}1\right)$; 39.6° $\left(10\bar{1}2\right)$; 40.4° $\left(11\bar{2}1\right)$; 42.4° $\left(20\bar{2}0\right)$; 45.8° $\left(20\bar{2}1\right)$; 50.08° $\left(11\bar{2}2\right)$; 54.9° $\left(20\bar{2}2\right)$; 59.9° $\left(21\bar{3}1\right)$; and 64.09°$\left(11\bar{2}3\right)$. Instrumental broadening was determined with a powdered quartz single crystal (recorded with the same measurement settings) and subtracted from our silcrete measurements at each degree 2*ϴ* before the W-H calculations. To interpret domain size from the plots, a shape factor *K* of 0.94, for a roughly cubic domain shape, was used.

#### Near infrared spectroscopy (NIR)

The infrared transmission of each polished slab was measured at normal incidence between 4000 and 5600 cm^−1^ with a resolution of 4 cm^−1^, using an Agilent Cary 660-IR FTIR spectrometer and unpolarised radiation. The infrared spectra of quartz show two bands in this region: (i) a SiOH combination mode involving O—H stretching and Si—O—H bending causes a band near 4600 cm^−1^; and (ii) a H_2_O (*ν*2 + *ν*3) combination band near 5200 cm^−1^ [39–41]. Baselines for absorbance measurements of the two combination bands were straight lines between the two lowest points on either side of the bands. All absorbances were measured directly on the IR spectra without smoothing or baseline subtraction. Error bars for the measured ‘water’ contents were calculated by repeating spectral acquisition and data treatment on one slab of each sample for ten times. For this, the slabs were mounted in the spectrometer, a spectrum was acquired, the slab was un-mounted and the procedure was repeated. The resulting error bars reflect the heterogeneity of the four silcrete samples in terms of OH, i.e. the within-sample variation of the concentration of different ‘water’ species.

We calculated the mass of silanol and molecular water using the molar absorption coefficients given in Scholze [39], and the formula and protocol given in Schmidt et al. [7]. The volume of intergranular pore space was also calculated with the protocol defined in this work: structural H_2_O measured in the Dehy-state was subtracted from the total H_2_O concentration measured in the Rehy-state to obtain a value corresponding to loosely held H_2_O adsorbed in the open pore space within samples. Pore volume was then calculated from the densities of H_2_O, quartz and anatase (TiO_2_) (the mass of TiO_2_ was taken from our geochemical analyses; see below). IR-measurements with this protocol result in three values: (i) the loss/gain of tightly held structural H_2_O at each heat treatment temperature in wt% of the silcrete sample (Dehy-measurements); (ii) the loss/gain of SiOH at each temperature in wt% (Dehy-measurements); (iii) the loss/gain of pore space in vol% of the rocks at each temperature (from the subtraction of Dehy- from Rehy-measurements). To plot heat-induced differences, values measured after heat treatment were subtracted from the control values recorded on the same polished slab before heat treatment. If no thermal transformations occurred after heating to a target temperature, all values will plot as 0. If one of the water-species or the pore space volume is lost, its value will plot as a negative value (likewise, gain will plot positive). ‘Water’ and porosity concentrations of the unheated samples correspond to the means of (i), (ii) and (iii) recorded in the nine sample slabs before heat treatment. Additionally, we recorded one near- to mid-IR spectrum between 5500 cm^-1^ and 2000 cm^-1^ of each sample slab to test for the presence of specific OH bands of clay (see for example [42]).

#### Geochemical analysis

Each of the pulverised samples underwent analysis by ICP-MS and ICP-AES to detect minor and trace element concentrations before and after heat treatment at different temperatures. Minor oxide and base metal concentrations were determined using a Varian 700 series ICP-AES instrument. Volatile, trace and rare earth element concentrations were determined using an Elan 9000 ICP-MS instrument. For sample preparation, a lithium borate fusion was used for the resistive elements, a four acid digestion for the base metals, and an aqua regia digestion for the volatile gold-related trace elements. Minor elements are reported as oxides in wt%; trace elements as atoms in ppm; loss on ignition was determined using a thermal decomposition furnace and is reported in wt%. All analyses are certified under quality control certificates SV11196510 and SV12059240 issued by ALS Minerals, Sevilla, Spain. Analysing the complete set of geochemical data lies beyond the scope of this study, and will be the subject of a separate publication on the geochemical changes arising from the heat treatment of silcrete. Here we report only elements that were found to be subject to change upon heat treatment.

Similar to the treatment of our IR data, we first subtracted element values measured on the heat-treated granular aliquots from the values of the unheated aliquots. This ‘loss’-value was then subtracted from the mean concentration of this element as averaged from the nine unheated aliquots. The resulting values are representative of the overall concentration of an element in a sample, displaying its loss after heating to different temperatures. Values are plotted with two error bars. The first corresponds to the instrumental error for the plotted element. The second (normally larger) error bar corresponds to the range of element concentration values as recorded in the nine not heat-treated granular aliquots from the same sample. This error reflects the heterogeneity of the sample with respect to the plotted element. Major and minor elemental compositions of the unheated samples in Table 2 correspond to the means of detected element percentages of all nine unheated sample aliquots from each original silcrete block.

**Table 2:** Major and minor element composition as determined by AES.

	WK-13-13	WK-15-01	BW-14-01	BW-14-02
**SiO**_**2**_	97.09	98.02	98.16	98.87
**Al**_**2**_**O**_**3**_	0.41	0.12	0.23	0.19
**Fe**_**2**_**O**_**3**_	0.38	0.10	0.31	0.23
**CaO**	0.04	0.03	0.03	0.02
**MgO**	0.02	0.01	0.03	0.01
**Na**_**2**_**O**	0.02	0.01	0.04	0.05
**K**_**2**_**O**	0.03	0.02	0.12	0.10
**Cr**_**2**_**O**_**3**_	0.00	0.01	0.01	0.01
**TiO**_**2**_	0.85	0.73	0.04	0.04
**MnO**	0.00	0.00	0.00	0.00
**P**_**2**_**O**_**5**_	0.02	0.01	0.00	0.00
**SrO**	0.00	0.00	0.00	0.00
**BaO**	0.02	0.02	0.02	0.02
**LOI (wt%)**	0.43	0.18	0.60	0.66

LOI = loss on ignition. Element values are in wt%.

## Results

### Description and comparison of samples

#### Macroscopic description

**Fig 2:**
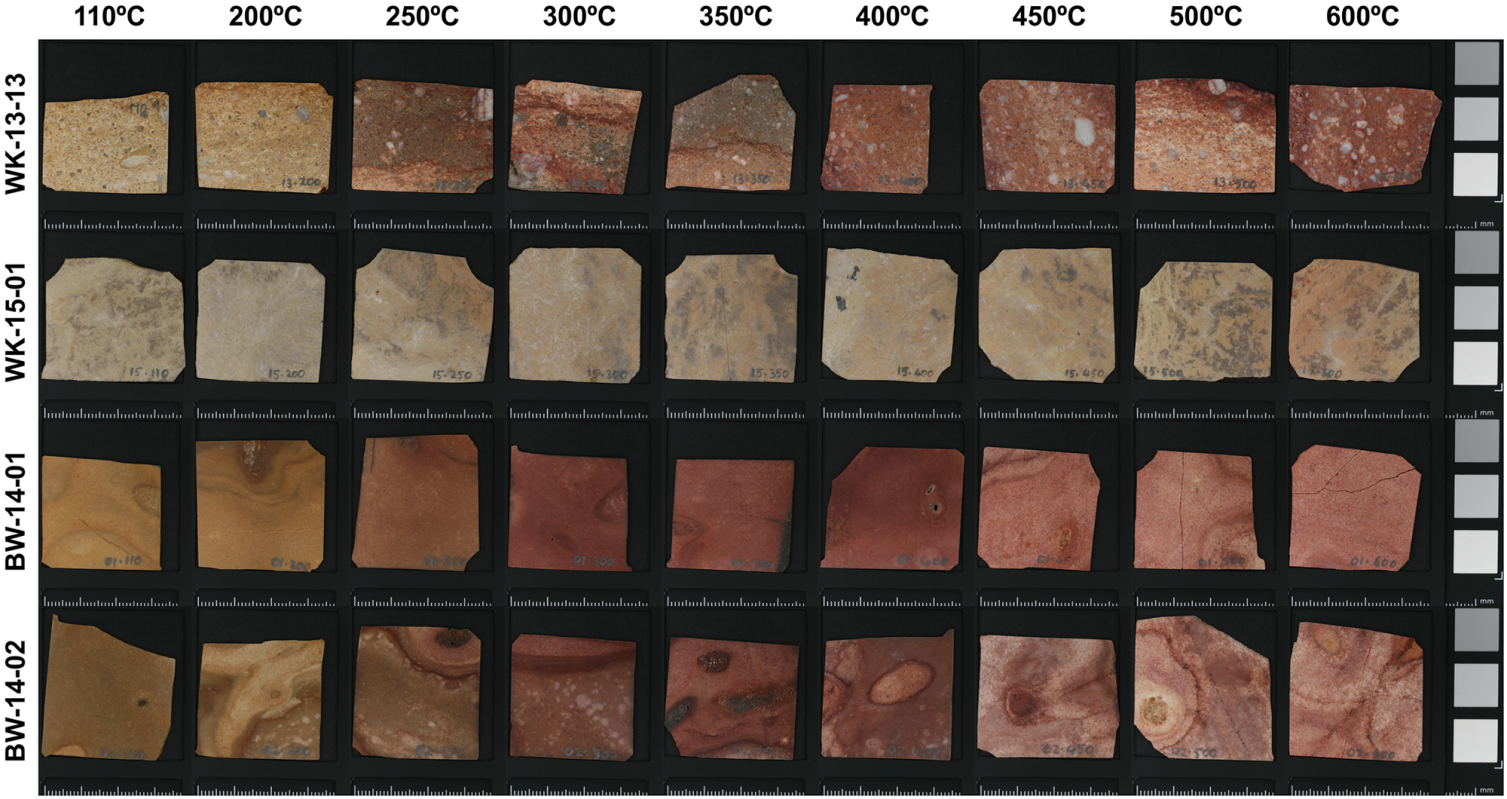
Thumbnail images of the polished slabs from each of the four silcrete samples (top to bottom) plotted against temperature stage (left to right). These are the samples used for colour change and IR-spectroscopic analyses. Scale bars in mm.

The two South African silcrete samples are both yellowish light-brown in colour and produce matt fracture surfaces. WK-15-01 has a finer texture, and quartz clasts are only visible with a hand lens (as can be seen from the images of the polished slabs in Fig 2). Prominent markers of illuviation are visible within the samples, including colloform features [43], a yellowish net of illuviation conduits, and clay skins. WK-13-13 contains large quartz clasts of up to a few mm in diameter (Fig 2). Irregular zonation between lighter and darker areas, and occasional colloform features, are visible. Both South African samples were collected from two *in situ* weathering profiles where silcrete horizons directly overly thick kaolinite deposits. This, and their macroscopic markers of sub-soil processes, confirms the two samples to be pedogenic silcretes. The two samples from Botswana are light brown in colour and produce shiny fracture surfaces. They cannot be distinguished based on macroscopic criteria. Abundant sub-circular to unshaped zones with rounded edges are visible all over their surfaces. Tightly packed quartz clasts can be distinguished with a hand lens. No markers of illuviation are visible. Both samples were collected from the floodplain of the Boteti River near Samedupi Drift, and have been interpreted by Shaw and Nash [22] as drainage-line silcretes, a variety of non-pedogenic silcrete [26].

#### Petrographic description

**Fig 3:**
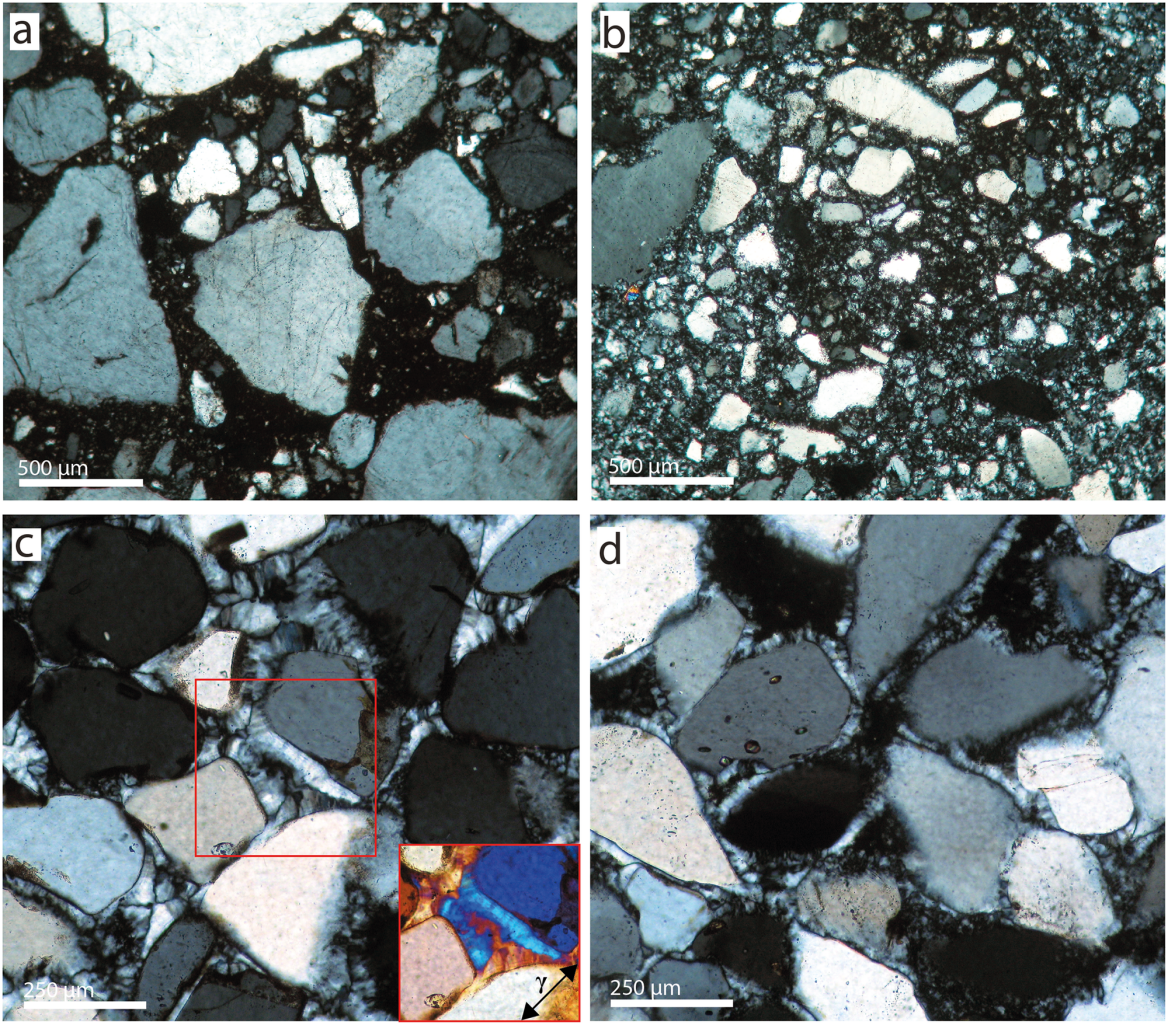
Thin section micrographs of the four silcrete samples. (a) WK-13-13, (b) WK-15-01, (c) BW-14-01, (d) BW-14-02. The two WK- samples are composed of quartz clasts and microquartz cement of varying crystal size. BW-14-01 (a) is composed of quartz clasts with epitaxial chalcedony overgrowth. Chalcedony fills the entire inter-clast space. The inset in the lower right of (c) is the area marked by the frame in the centre of the micrograph with a 1 λ full waveplate (orientation of the waveplate’s higher refractive index (*γ*) is arrowed). The addition of the waveplate’s *γ* and the chalcedony’s fibre axis to blue reveals the latter to be Length-slow (LS) chalcedony. BW-14-02 is composed of quartz clasts with epitaxial LS-chalcedony overgrowth, but the centres of the inter-clast voids are filled with a cement composed of length-fast chalcedony. (a, b) 10x dry objective, (c, d) 20x oil immersion objective.

Microscopically-determined clast to matrix percentages and grain sizes are summarised in Table 1. The South African samples both consist of quartz clasts in a microquartz matrix. WK-13-13 (Fig 3a) has a floating (F-)fabric [43], consisting of fine matrix grains and large clasts (mean grain size ~0.92 mm), some of the latter being close enough together to be described as a grain-supported (GS-) fabric [43]. Clasts are angular, unsorted and exhibit dissolution features on their surfaces. The matrix is almost opaque in unpolarised light, revealing higher concentrations of non-quartz components than in WK-15-01. WK-15-01 (Fig 3b) also has an F-fabric, consisting of angular and unsorted clasts (with an average grain size of ~0.2 mm), floating in a matrix of medium to fine microquartz. Distinguishing between large matrix grains and small clasts is difficult because of their similarity in size. In unpolarised light, the matrix is brown in colour, with a few μm-sized pellets (most likely anatase, see [43]) visible at highest magnification. The samples from Botswana both have a GS-fabric consisting of rounded well-sorted clasts (mean grain size ~0.25 mm) with occasional dissolution bays and a matrix of chalcedony (Fig 3c and 3d). The matrix is composed of a layer of varying thickness of epitaxial length-slow (LS) chalcedony overgrowth on the clasts. Larger voids are filled with length-fast (LF) chalcedony. Both samples are petrographically very similar, with the main difference being that the LF-chalcedony filled voids are typically larger in BW-14-02. The matrix in both samples is transparent in unpolarised light, suggesting a low concentration of non-quartz impurities. No microscopic pore space is visible in any of the four samples.

#### Mineralogy, structure and chemistry of the samples

The chemical composition of the four samples is summarised in Table 2. All samples comprise >97% SiO_2_. The two South African samples contain slightly more non-SiO_2_ components than the samples from Botswana. These are mainly Fe_2_O_3_ and TiO_2_ for the South African samples and Al_2_O_3_, Fe_2_O_3_ and K_2_O for the Botswana samples. Powder diffraction data from the four unheated sample powders suggest that the higher TiO_2_ concentration within the two South African samples is due to the presence of anatase. Diffraction data for the Botswana samples indicate only the presence of quartz. The mid-IR spectrum of sample WK-13-13 shows traces of a 1:1 clay (possibly kaolinite) present as an impurity with in the silcrete (S4 Fig). None of the other mid-IR spectra indicate the presence of clay minerals.

The two South African samples contain 0.36–0.56 wt% of ‘water’ (composed of SiOH and H_2_O); the samples from Botswana contain slightly more ‘water’, in the range 0.54–0.89 wt%. These water concentrations are slightly higher than the mass lost on ignition, except for one sample (compare Tables 2 and 3). This can most likely be explained by some SiOH being retained up to elevated temperatures [40], as well as minor imprecisions of Scholze’s [39] specific absorption coefficients of ‘water’ when applied to silcrete [7]. Additionally, the measured loss on ignition values may be affected by the decomposition of organic phases and the oxidation of amorphous carbon, further complicating the relation between this value and the ‘water’ content of the samples. The South African samples have approximately three times more pore space (mean of 2.12 vol%) than the Botswana samples (mean of 0.62 vol%).

**Table 3:** Results of the XRD analysis of internal crystal strain and size of the coherent scattering domain.

	WK-13-13		WK-15-01		BW-14-01		BW-14-02	
Temp. (°C)	Strain (%)	Size (nm)	Strain (%)	Size (nm)	Strain (%)	Size (nm)	Strain (%)	Size (nm)
**110**	0.0196	233.6	-0.0083	124.8	-0.0349	44.3	0.031	254.1
**200**	0.0146	289.6	-0.0094	123.8	-0.0054	70.0	0.0243	289.6
**250**	0.0171	329.1	-0.0054	152.4	-0.0069	95.3	0.018	166.5
**300**	0.0162	206.9	-0.0133	88.3	0.0082	181.0	0.0262	229.9
**350**	0.0247	249.7	-0.0046	185.7	-0.0028	135.3	0.0309	108.9
**400**	0.0215	209.9	-0.0195	92.2	-0.0189	63.2	0.0391	314.8
**450**	0.0318	321.8	-0.0262	69.3	-0.0024	65.2	0.0297	249.7
**500**	0.0212	289.6	-0.0034	121.7	-0.0219	68.6	0.0231	188.1
**600**	0.0417	314.8	-0.0092	114.0	-0.0217	69.3	0.0243	213.0

Williamsons-Hall plots of the diffraction data for unheated samples (S5 Fig) provide data on the mean internal strain of the quartz crystals in each sample. However, it should be borne in mind that these strain values are not strictly representative of the matrix-grains, because *à priori* unstrained clasts are also taken into account by the bulk rock analysis. As such, they provide only a qualitative estimation of whether or not matrix grains are subject to internal strain. Both South African samples yielded strain values of 0.02–0.03%, while the Botswana samples show slightly negative to no strain.

### Thermal transformations

#### Colour change upon heat treatment

Thumbnail images of each of the 36 polished silcrete slabs used for colour change and IR-spectroscopic analyses are shown in Fig 2. Pronounced reddening after heating to 200°C can be observed visually in three of the four samples (WK-13-13, BW-14-01 and -02). The most intense reddening in these samples is associated with zones/bands of higher iron oxide content in the initial polished slab, with areas of more uniform silica cement or matrix showing less intense, but nonetheless marked, colour changes. This leads to the visual effect that some larger zones on the sample blocks do not redden while others do (Fig 2). In the polished slabs, visual intensity of redness appears to decline after 450°C in samples BW-14-01 and -02, but is maintained in sample WK-13-13. Sample WK-15-01 has a lower initial iron oxide content, but becomes noticeably pinker in colour after heating to around 200°C, with the intensity of pinkness maintained at higher stages of heating.

**Fig 4:**
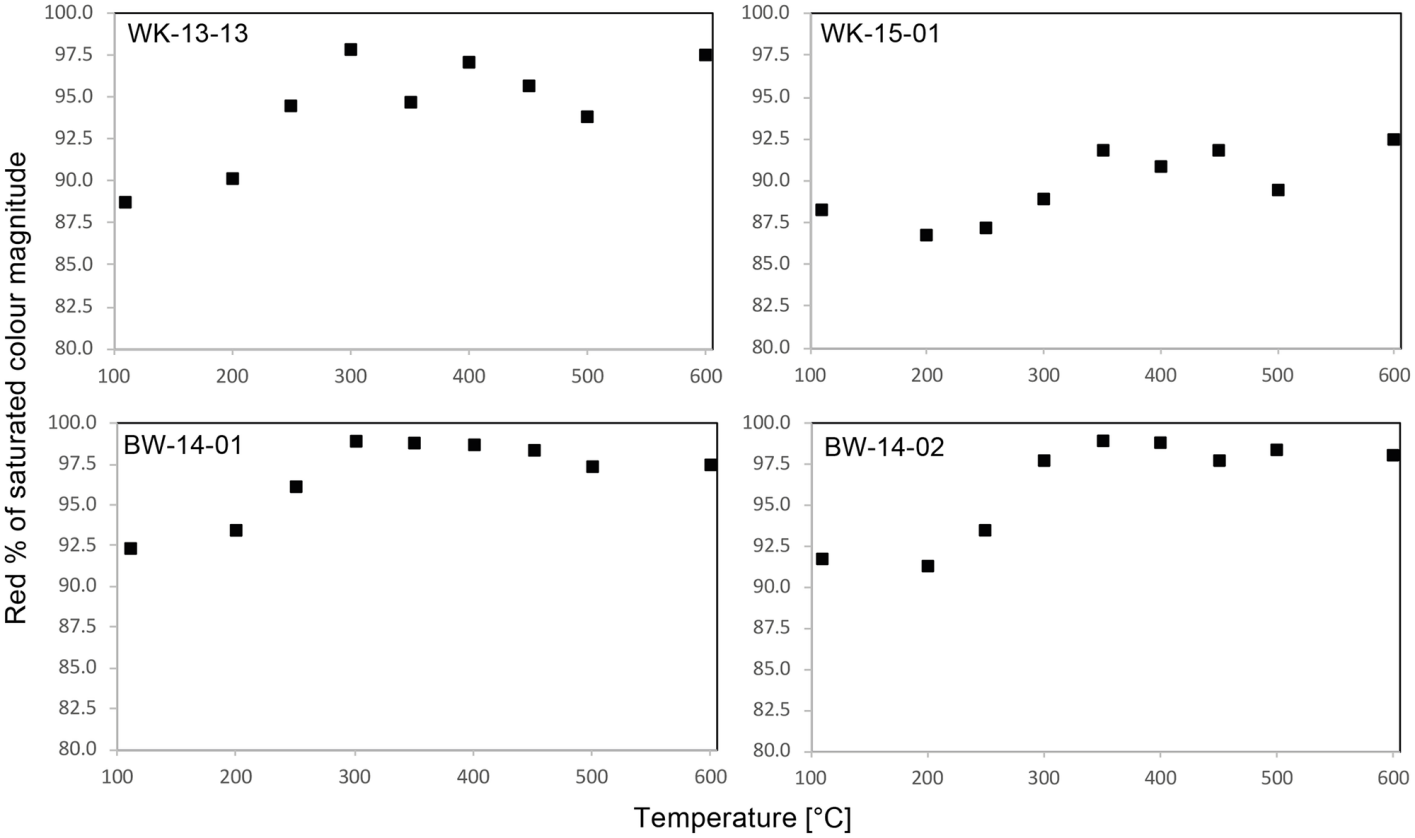
Plot of the temperature-dependent change in saturated red reflectance as a percentage of the saturated colour magnitude.

These visual trends in colour change are verified by the results of more objective image analysis (Fig 4). The samples from Botswana have a higher initial saturated red reflectance as a percentage of the saturated colour magnitude (henceforth ‘Red%’) compared to the two Cape silcrete samples—i.e. they are initially redder than the samples from the Cape. However, samples WK-13-13, BW-14-01 and -02 exhibit a similar 7–9% shift in Red% after 200°C, all reaching maximum redness after 300°C. For sample WK-15-01, the increase in redness is apparent at a slightly later stage of heating, reaching maximum Red% after 350°C. The decline in redness apparent visually in samples BW-14-01 and -02 in Fig 2 is much less obvious in Fig 4, with only a 1–2% decline in Red% after 450°C.

#### Crystallographic change

**Fig 5:**
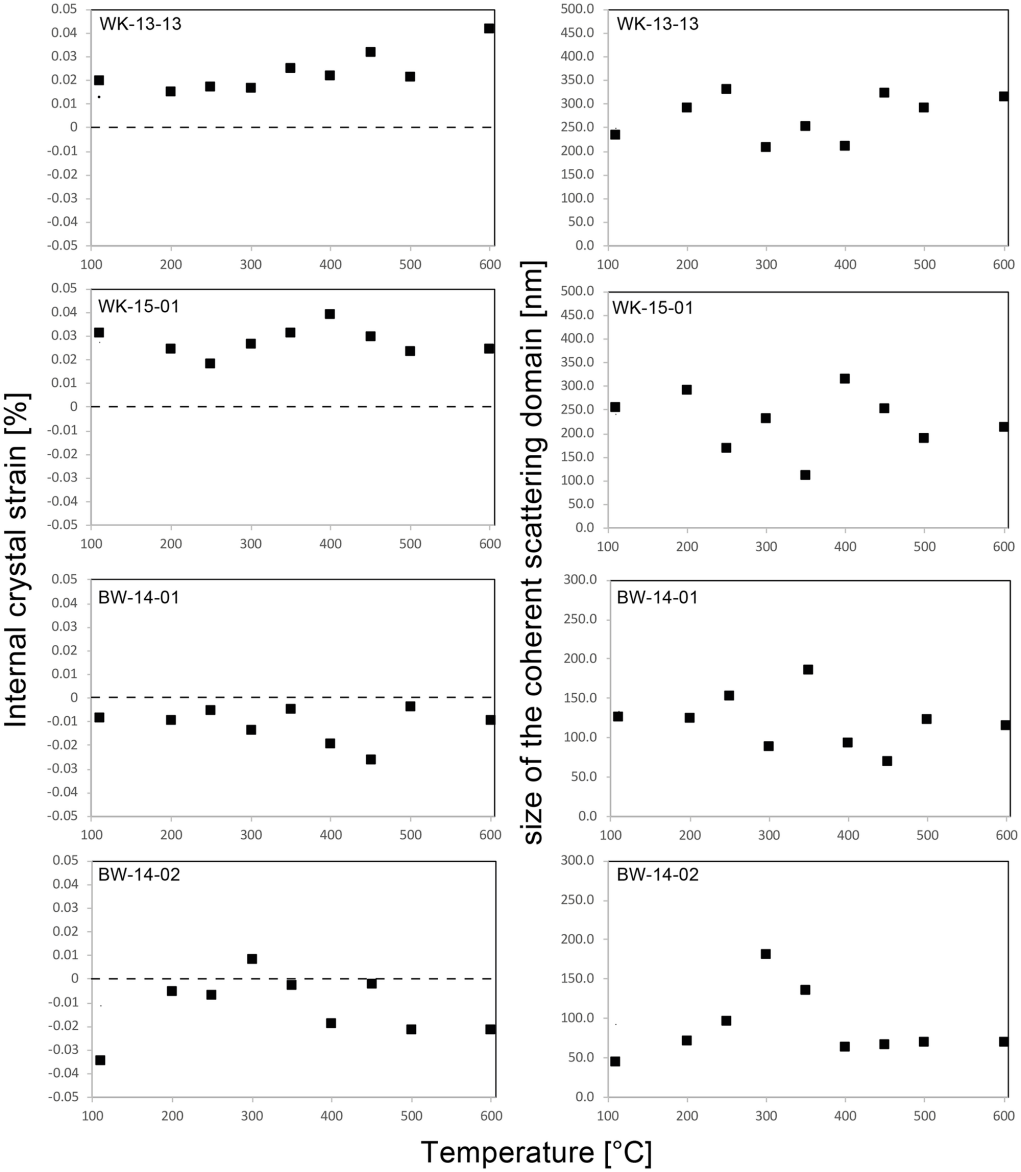
Plot of the evolution of internal strain in percent and average size of the coherent scattering domain in nanometre upon heat treatment. Values were determined by Williamsons-Hall plots. Note that no clear thermal evolution can be observed in any of the samples.

XRD data are summarised in Table 3. Fig 5 is a plot of the evolution of internal strain and average grain-size upon heat treatment, as determined by Williamsons-Hall plots. Across all temperature steps, both samples from Botswana show slightly negative internal strain values (although globally close to 0), whereas both samples from South Africa show positive values. Average size of the coherent scattering domain is also smaller in samples from Botswana. Most of the temperature-dependant size and strain plots of the four samples show no discernible evolution. The range of values recorded at different temperatures appears to be caused by sample heterogeneity or measurement imprecision. The only two exceptions are the internal strain values recorded in South African sample WK-13-13, showing steadily increasing values upon heat treatment, and domain size data recorded in sample BW-14-02, which may be interpreted to show increasing domain sizes up to 300°C followed by decreasing size at higher temperatures.

#### ‘Water’-related and structural transformations

**Fig 6:**
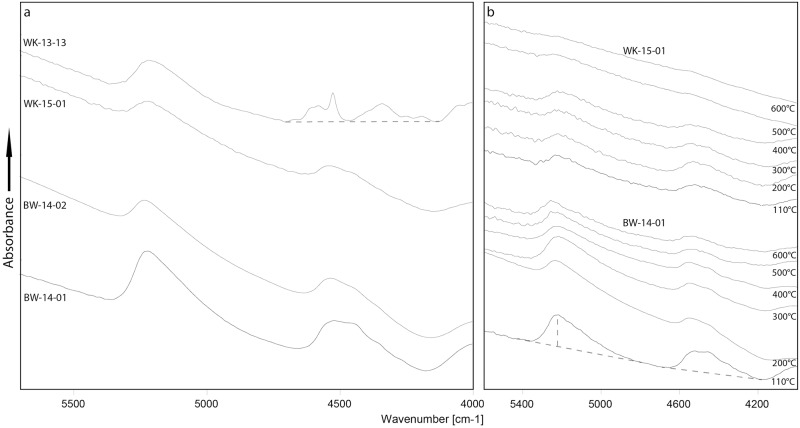
Near infrared spectra of the four silcrete samples. (a) Spectra of the four samples before heat treatment. Each spectrum is the average of the nine spectra recorded from the polished slabs before the heating experiment, representing an average of the initial block. The left band near 5200 cm^−1^ is caused by H_2_O and the right band near 4600 cm^−1^ by chemically bound water (SiOH). Note that WK-13-13 has a slightly different spectrum in the SiOH region. The sharp band results from OH in a 1:1 clay mineral present in the sample matrix as an impurity. (b) Temperature-dependant evolution of the spectra of WK-15-01 (upper part) and BW-14-01 (lower part). Note the gradual loss of H_2_O and SiOH with increasing temperature. Baselines for the measurements are shown as broken lines. The baselines in (b) were used for all samples except for SiOH measurements in WK-13-13 (baseline in a). All spectra are recorded in the dehydrated state and displaced vertically for better readability.

**Fig 7:**
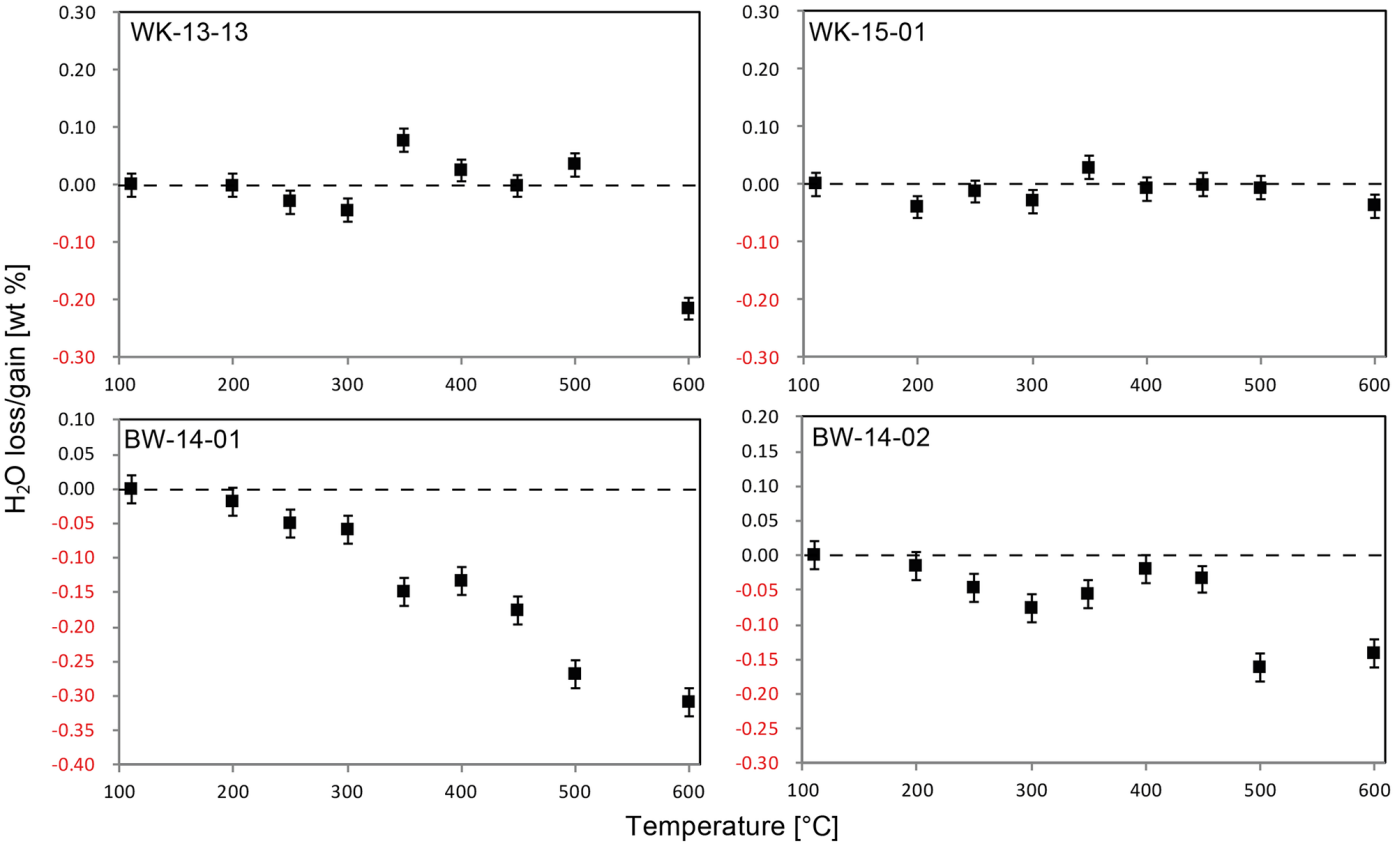
Heat-induced loss of structural H_2_O in the four silcrete samples. Note that the two South African samples (top) show no discernible water loss/gain. Both samples from Botswana (bottom) lose water from 200°C onwards.

**Fig 8:**
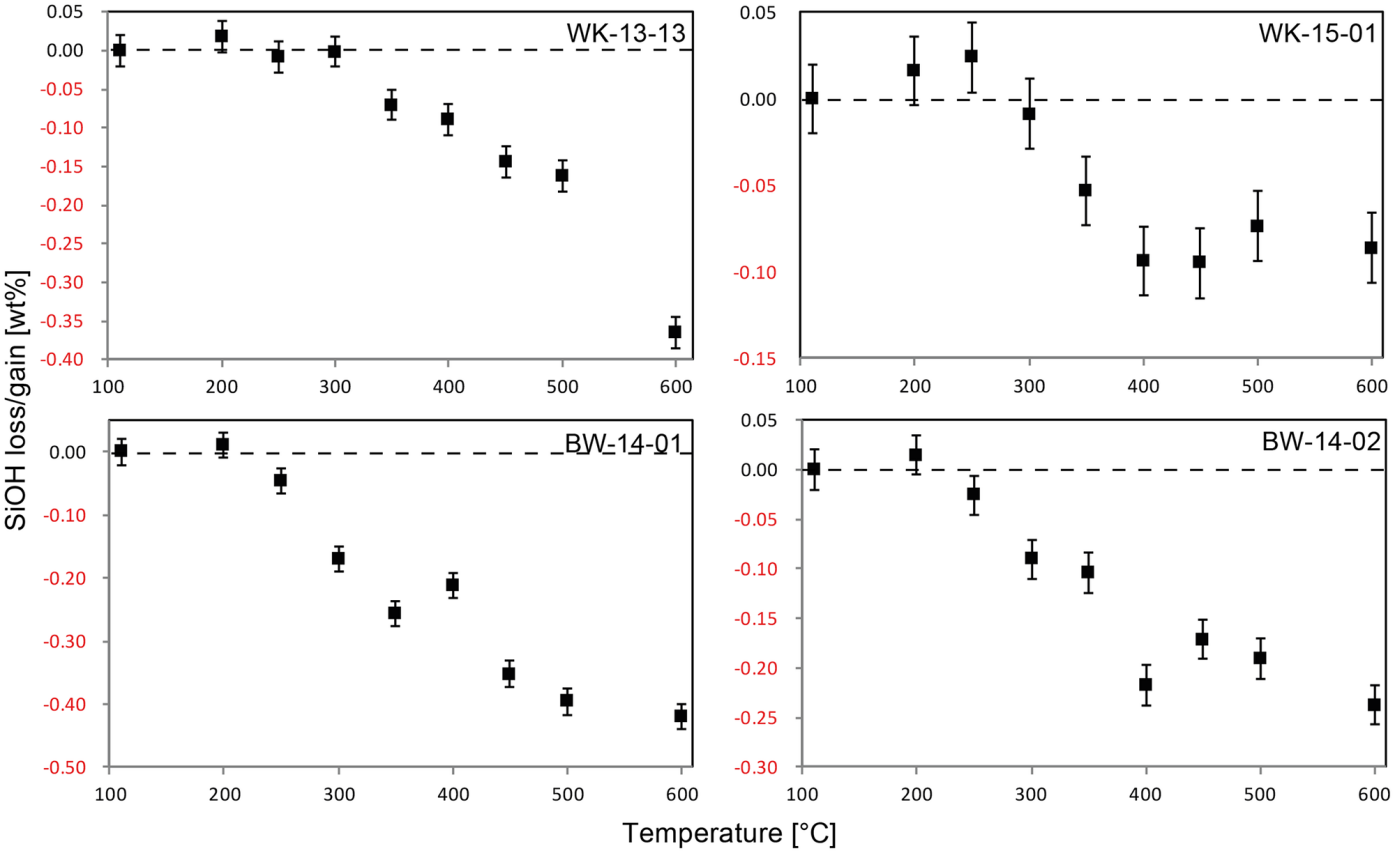
Heat-induced loss of SiOH in the four silcrete samples. Note that the two South African samples (top) begin to lose structurally bound water from 350°C upwards. Both samples from Botswana (bottom) lose SiOH from 200°C upwards, hence 150°C lower than the onset of the reaction in South African silcrete.

Fig 6 shows NIR spectra of the four samples prior to heat treatment, and the temperature dependant evolution of the spectra of two of the samples (WK-15-01 and BW-14-01). Both samples from Botswana and sample WK-15-01 from South Africa yielded spectra comparable to previously published data [7, 8], suggesting that molecular and chemically-bound water are held at similar structural sites. WK-13-13 yielded a significantly different spectrum in the SiOH region, documenting the presence of 1:1 clay (see also S4 Fig). However, integrating the whole SiOH absorption envelope (cf. baseline in Fig 6a) still provides data on the relative temperature-induced loss of chemically-bound water in the bulk sample. Fig 7 incorporates plots of the loss of molecular water strongly held in the structure of each sample. The two South African samples show no discernible water loss or gain. Both samples from Botswana, however, lose water from 200°C onwards; after heating to 600°C, approximately 56% and 58% of the total H_2_O content are lost in BW-14-01 and -02 respectively. A similar trend is seen in the SiOH plots for all four samples (Fig 8). The two South African samples begin to lose SiOH from 350°C upwards; both Botswana samples lose SiOH from 200°C. Thus, the onset of the reaction lies approximately 150°C lower in the samples from Botswana compared to those from South Africa.

**Fig 9:**
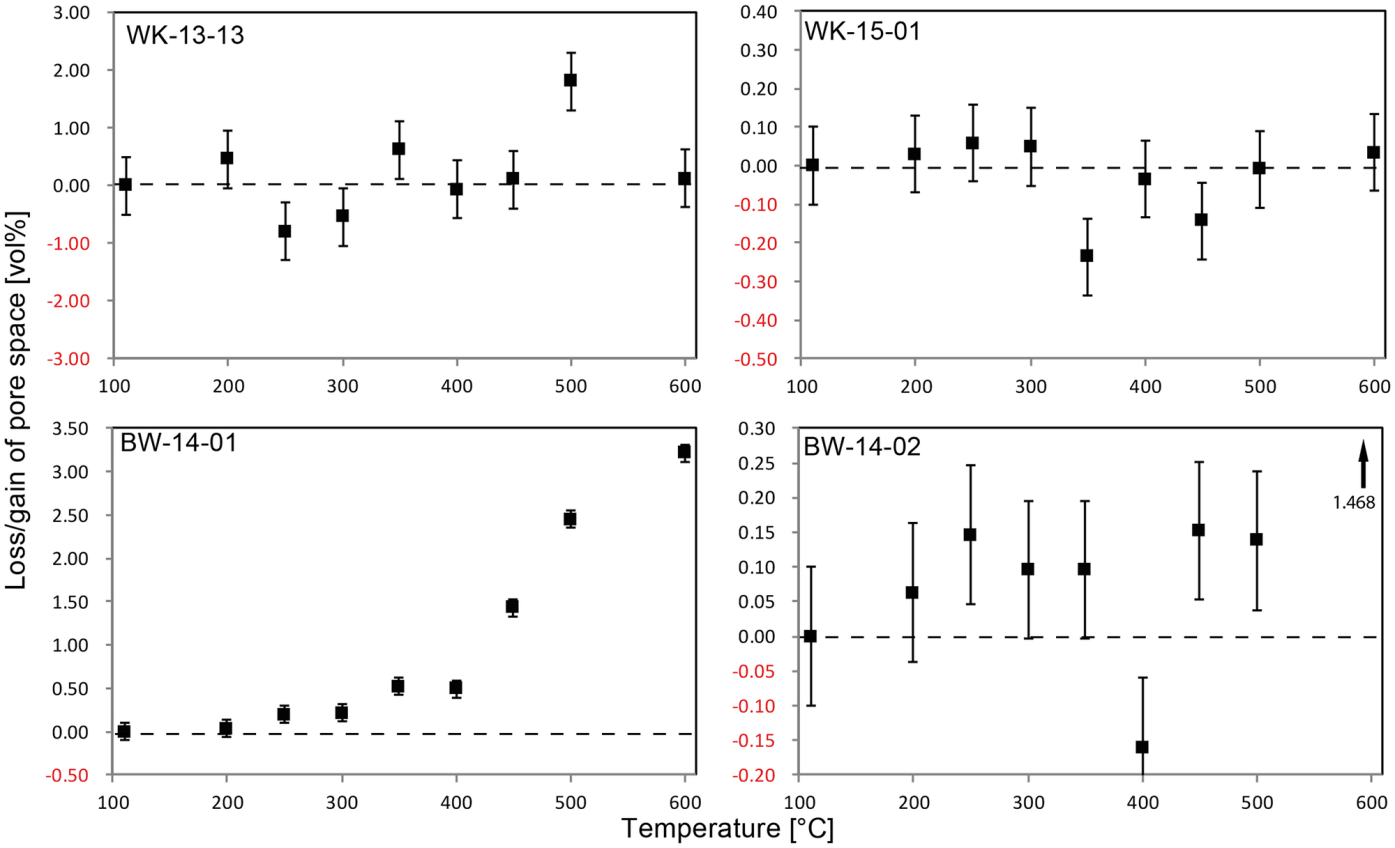
Heat-induced evolution of pore space in the four samples. Note that in the two South African samples (top) no clear trend is discernible. The porosity of both samples from Botswana (bottom) increases from 200°C upwards.

The thermal evolution of the porosity of samples shows further differences between samples from Botswana and the Cape (Fig 9). No clear trend in pore space is discernible in the two South African samples. However, the porosities of both Botswana samples increase from 200°C onwards. After heating to 250°C, the total pore spaces within BW-14-01 and -02 are relatively increased by 33% and 31% respectively. Heating to higher temperatures further increases pore space (450°C: 165% for BW-14-01 and 40% for -02; 600°C: 241% for BW-14-01 and 365% for -02).

### Other chemical transformations

**Fig 10:**
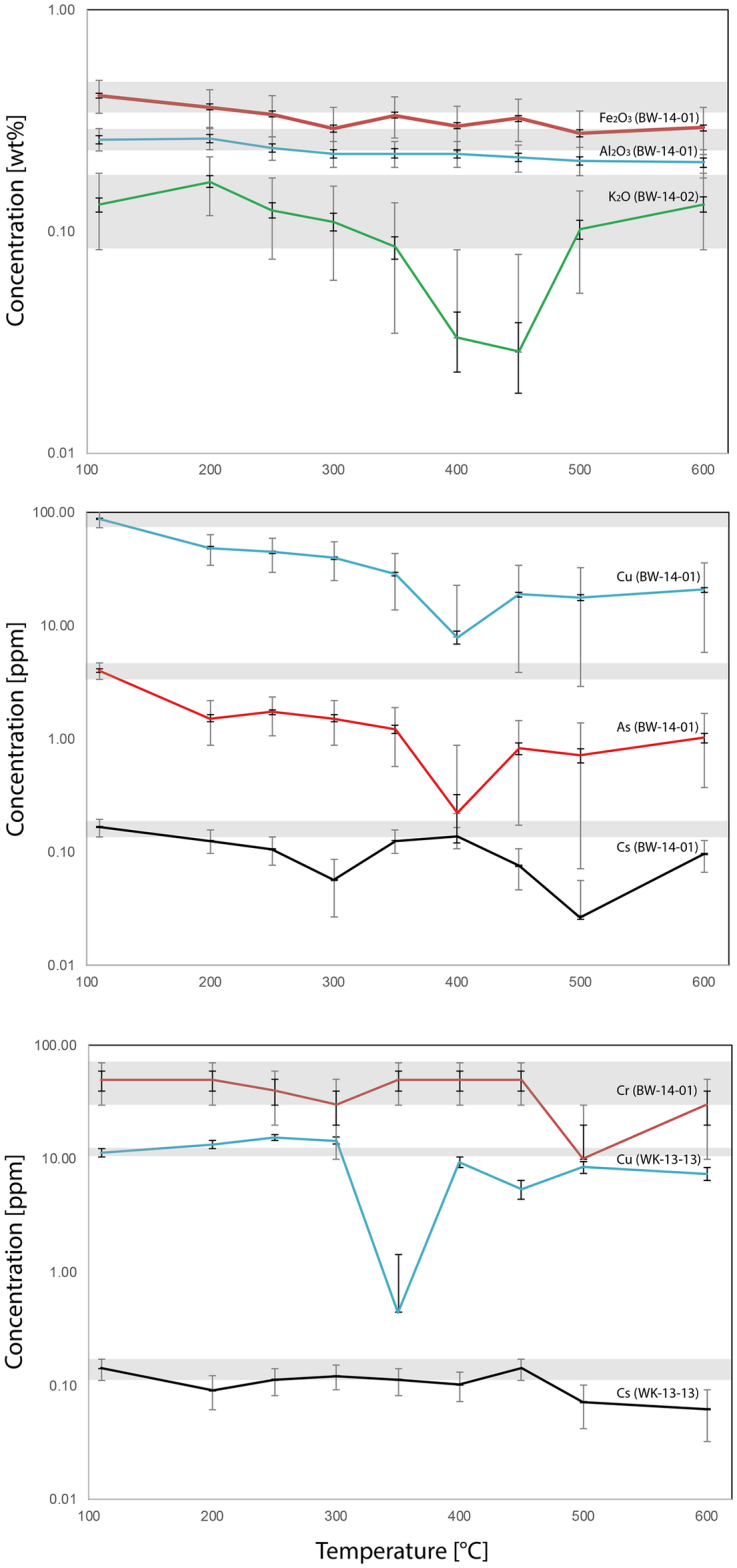
Plots of the concentration of minor (top) and trace elements (lower two graphs) in the three samples exhibiting element depletion. Grey horizontal bars mark the range of error of the unheated sample (large error bars) and correspond to the sample’s heterogeneity with respect to the plotted element. Only values plotting below this range can confidently be considered as element depletion. Smaller error bars mark the instrumental error.

We found seven elements in three samples to be changed after heat treatment (Table 4). These are, minor elements: Al_2_O_3_ (in BW-14-01 only), K_2_O (in BW-14-02 only) and Fe_2_O_3_ (in BW-14-01 only); trace elements: Cr (in BW-14-01 only), Cu (in BW-14-01 and WK-13-13), As (in BW-14-01 only) and Cs (in BW-14-01 and WK-13-13). Significantly, no consistent patterns were found across all four samples. Depletion plots are summarized in Fig 10. Both minor elements Al_2_O_3_ and Fe_2_O_3_ are clearly depleted (i.e. plot outside the expected error) in granulars from sample BW-14-01 heated above 500°C, although the depletion trend seems to begin at lower temperatures. K_2_O is depleted in the two granulars from sample BW-14-02 heated to 400°C and 450°C. Cu, As and Cs in BW-14-01 are depleted in almost all heated granulars, indicating that these three trace elements are particularly susceptible to depletion in this sample. Cu in the South African sample WK-13-13 is depleted in all granulars heated above 350°C, and Cs in the two granulars heated above 500°C.

**Table 4:** Porosity, H_2_O and SiOH values (with error range) as determined by IR-spectroscopy, and the minor and trace elements depleted upon heat treatment.

	Porosity (vol%)	H_2_O (wt%)	SiOH (wt%)	H_2_O+SiOH (wt%)	Element depletion
**WK-13-13**	**2.55** +2.44, -1.56	**0.25** +0.15, -0.09	**0.31** +0.23, -0.21	**0.56**	Cu, Cs
**WK-15-01**	**1.69** +0.84, -0.83	**0.17** +0.07, -0.06	**0.19** +0.12, -0.06	**0.36**	-
**BW-14-01**	**0.83** +0.52, -0.40	**0.46** +0.09, -0.18	**0.43** +0.10, -0.17	**0.89**	Al_2_O_3_, Fe_2_O_3_, Cu, As, Cs, Cr
**BW-14-02**	**0.41** +0.12, -0.13	**0.23** +0.09, -0.05	**0.31** +0.07, -0.10	**0.54**	K_2_O

‘Water’ content and porosity values were averaged from the nine unheated granular aliquots of each sample. Errors were determined as the maximum and minimum values recorded from the nine aliquots and reflect sample heterogeneity in terms of H_2_O, SiOH and porosity.

#### Thermal transformation of fracture patterns

**Fig 11:**
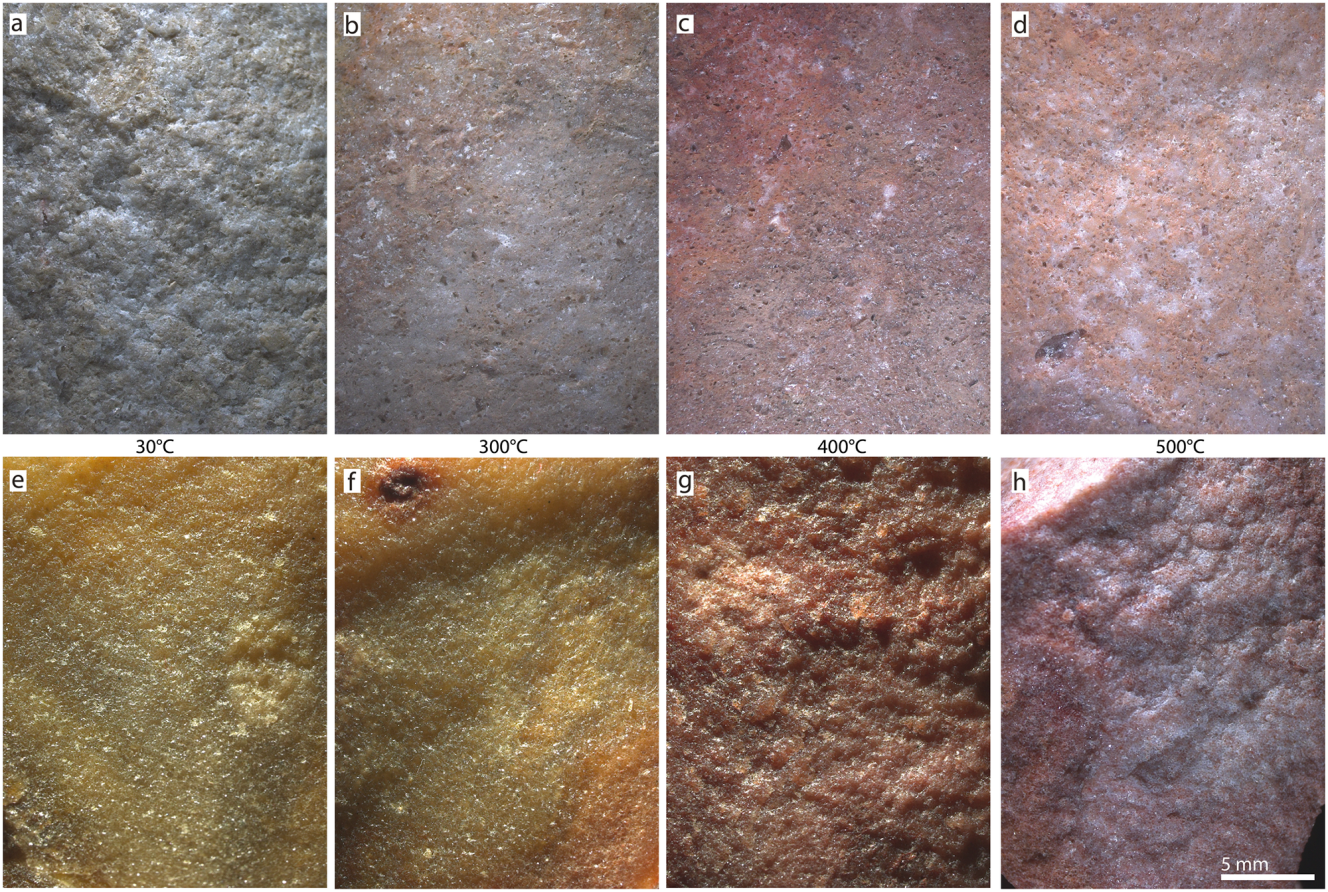
Results of the qualitative evaluation of fracture patterns on fresh removal scars before and after heat treatment. (a-d): Cape silcrete WK-15-01; (e-h): Kalahari silcrete BW-14-02. Heating temperatures between two photos apply to both silcrete types. All photos were taken at the same magnification and under identical raking light conditions. Note that while the Cape silcrete (top row) produces smoother fracture surfaces after heat treatment, the inverse trend is observed on the Kalahari silcrete (bottom row).

Experimental heat treatment of the two samples from South Africa and Botswana reveal further differences between both silcrete types. Fig 11 compares macro-photos of the fracture surfaces on BW-14-02 and WK-15-01, heat-treated with different temperatures. Freshly knapped surfaces on the South African sample become smoother after heat treatment at 300°C and 400°C, indicating the effectiveness of the thermally-induced chemical reaction. Only heating to 500°C leads to a fracture pattern creating slightly rougher surfaces than heating to lower temperatures. We observed no heat-induced fracturing of WK-15-01. This trend cannot be observed on the sample from Botswana. Heat treatment at 300°C does not visually transform the sample’s fracture pattern, and freshly knapped surfaces are indistinguishable from surfaces knapped before heat treatment. Heat treatment at 400°C resulted in heat-induced fracturing of the block. When we knapped the remaining fragments, the fracture pattern was significantly rougher than before heat treatment. Knapping of BW-14-02 after heat treatment at 500°C produced an even rougher fracture pattern. Thus, while the knapping quality of our South African silcrete sample was improved by heat treatment at 300°C and 400°C, and this trend was only inverted at higher temperatures, the Botswana sample was not improved by heat treatment. Indeed, knapping quality noticeably deteriorated from 400°C onwards.

## Discussion

### A critical review of IR and XRD data

The type of data we present here is new, and our experimental approach has never been applied to silcrete or any other silica-rich rock. Although similar values on the evolution of H_2_O, SiOH and porosity exist, the inherent differences of our experimental setup must be borne in mind when interpreting the resulting data. Previous studies by the lead author on heat treatment of silcrete [7, 8] and flint [34, 44] relied on a sequence of successive heating steps applied to the same IR sample slab. Here we measured ‘water’ and porosity on several IR sample slabs cut from different parts of the sample block, each heated separately to different temperatures. Although thermal transformations were shown to be the same during heating sequences and single heating [33, 34, 45], sample heterogeneity can be expected to play an important role here. While our protocol was designed to minimise the heterogeneity between IR- and geochemistry-samples, it introduced heterogeneity between different IR-samples from a single original silcrete block (cf. calculated errors in Table 3). This effect can be seen from the ‘noise’ in our data on porosity and H_2_O evolution in both South African samples (Figs 7 and 9). The thermal evolution of these two values were shown to be of weak magnitude in Cape silcrete [7], and our experimental approach did not produce values fine enough to resolve them. The experiment has, however, produced sufficiently fine values to determine the thermal evolution of SiOH in all samples, and porosity/structural H_2_O in samples from Botswana. Thus, all values from the two Kalahari silcrete samples are known and can be compared with silcrete from the Cape (also taking into account [7]). A similar effect can be observed in our XRD data on the evolution of the crystallography of samples. It has previously been shown for flint [44] that crystallographic parameters are subject to change. The magnitude of these transformations is small in flint and our experiments have not revealed any change in silcrete. The reason for this may also be sample heterogeneity. Whether the absence of change is real, or if there are weak thermal transformations of internal crystal strain or size of the coherent scattering domain in silcrete, as shown for flint, must be investigated by future studies relying on *in situ* measurements at successive temperatures. The only secure observation stemming from our data is that if there are thermally-induced crystallographic transformations, they are of low magnitude.

### Steam leaching: A newly recognised mechanism in the thermal transformations of silcrete

Another novelty of this study is the analysis of the thermal evolution of minor and trace elements arising from heat treatment. We observed the depletion of seven elements in three of our samples, although no elements were consistently depleted across all four samples. Except for Cs and As, these depleted elements are not volatile, and their temperature-induced loss must be explained by an alternative mechanism. Table 3 shows these elements and compares them with the overall porosity, and the molecular- and chemically bound water concentrations of samples (as averaged from the nine unheated granular aliquots of each original sample). From this comparison, it can be seen that six elements were depleted in Botswana sample BW-14-01. This sample also contains the highest concentration of ‘water’. Two elements were depleted in WK-13-13, with the second highest ‘water’ concentration; one element was depleted in BW-14-02 with the third highest ‘water’ concentration. We measured no element depletion in WK-15-01, which had the lowest ‘water’ concentration. These data suggest a relationship between element depletion and the activity of water in the samples during heat treatment.

A further, weaker, relationship can be seen with the pore space volume of individual samples. Both Botswana samples have less pore space relative to their ‘water’ content (i.e. (H_2_O+SiOH)/Porosity = 1 in BW-14-01 and 1.3 in -02, against 0.2 in both South African samples). If the lower range of pore space values is considered (i.e. the lower error in Table 3), BW-14-01, in which six elements were depleted, has the least pore space relative to its ‘water’ content. This relationship can be explained by water leaching out some of the elements during heat treatment. Part of the molecular water released during the reaction

$$\text{Si}-\text{OH}\cdots \text{OH}-\text{Si }\to \text{ Si}-\text{O}-\text{Si }+{\text{ H}}_{2}\text{O}$$ (1)

taking place in silcrete during heat treatment [7], is expected to form steam. This steam phase can be expected to build up high pressure [46], particularly in samples with much water and little pore space. In a study on chalcedony, Pelto [32] showed that these rocks are particularly susceptible to steam leaching, and that steam created during heat treatment dissolves part of the quartz at the surface of pore walls. When applied to our silcrete samples, this suggests that Pelto’s mechanism may leach out some of the minor and trace elements trapped in quartz grains that are near pores. Our results support this model of steam-related depletion of some elements in silcrete during heat treatment. Whether chalcedony is more prone to such steam leaching than microquartz cannot be decided on the basis of our results. As it stands, heat treatment does appear to affect the minor and trace element composition of silcrete, although not all elements seem to be affected by steam leaching and there is no coherent pattern between samples. Future systematic studies should shed light on the mechanisms behind steam leaching and its effective conditions.

### A comparison between Cape and Kalahari silcrete

Our results confirm that, although the silcrete samples from the Cape and the Botswana Kalahari used in this study are chemically similar, being almost pure silica, they are very different in terms of their petrography, crystallography and mode of genesis. Most significantly, the cement or matrix of the Botswana silcrete is composed of LS-chalcedony overgrowths and LF-chalcedony void fills, whereas the South African samples are dominated by microquartz. These contrasting silica cements reflect different conditions during silcrete formation, and are likely to imbue the silcretes with different properties during their utilisation as tool stones. The differences between the two matrix types are further highlighted by our measurements of internal crystal strain and size of the coherent domain. The South African samples have a slightly positively strained matrix, while the crystallites in the silcretes from Botswana are almost not at all, to slightly negatively, strained. Average domain size in our Kalahari samples is also significantly smaller due to the presence of nanometre-sized crystallites in the chalcedony cement. These differences would be expected to affect the fracture pattern of unheated samples.

Our experimental investigation of fracture patterns shows clearly that removal scars on unheated Botswana silcrete samples are shinier than those on samples from South Africa (compare images (a) and (e) on Fig 11). Indeed, an observer familiar with South African silcrete could mistake the shiny fracture surfaces on unheated Kalahari silcrete as evidence of heat treatment (for surface gloss on heat-treated silcrete, see for example [3, 9, 11, 12]). Cape silcrete also contains a slightly lower but comparable amount of ‘water’, more impurities and a significantly larger pore space than Kalahari silcrete.

Overall, while grouping our samples under the term ‘silcrete’ makes sense from a broad geological perspective, the implied generalisation of rock properties accompanying this classification is dangerous for archaeologists seeking to analyse these materials in terms of a distinct material type used in the manufacture of tool stones. Much better would be to consider the geomorphological context within which the silcrete formed—and specifically whether or not it developed due to pedogenic processes—as this appears to have the greatest influence upon the material properties.

### Thermal transformations in Kalahari silcrete and their implications for knapping quality

Samples from both the Cape and Kalahari exhibited at least some reddening upon heating (Fig 2), indicating the oxidation of iron-rich inclusions (see for example [47]). However, the overall low concentrations of Fe_2_O_3_ make it unlikely that this phenomenon significantly contributes to the thermal transformations relevant for stone knapping. In terms of these thermal transformations, perhaps the most important difference between our samples is the earlier onset of the dehydration reaction in the Botswana silcretes when heated. This can be explained by the different crystallography of the two sets of samples; the matrices of the two Cape samples are microquartz, whereas the Botswana samples are cemented by LF- and LS-chalcedony. It has been shown previously [8] that chalcedony, as in flint and most marine chert, has a higher ‘water’ content than most microquartz in silcrete (compare [7] and [34]). Our results support this by showing that the chalcedony-rich Kalahari samples have higher water contents than microquartz-bearing Cape samples, even though the Kalahari samples have lower matrix to clast ratios. While the thermally-induced chemical reaction of chalcedony and microquartz is the same (reaction 1), it is initiated at higher temperatures in microquartz (again compare [7] and [34]). This is supported by our results.

Another difference is the creation of new pore space upon heating in the two Botswana samples. Our data on pore space in the two Cape samples is low resolution. However, no porosity increase upon heating has so far been identified in silcrete from the Cape coastal zone [7, 8]. Increasing pore space in silica rocks upon heating was previously assigned to internal fracturing [7, 8, 34, 44, 48, 49]; its dramatic increase in our Kalahari samples suggests intense fracturing upon heat treatment at relatively low temperatures. The mechanism behind such fracturing is the critical steam pressure of trapped H_2_O [46, 49] that is produced by reaction (1) and that cannot be evacuated through open pores [50]. Internal fracturing in our Kalahari samples is further supported by the loss of H_2_O from 200°C onwards (Fig 7), i.e. H_2_O, which below 200°C was tightly held in the rock structure, can be evacuated through newly formed cracks above 200°C.

Most LF-chalcedony-bearing rocks (i.e. flint and marine chert) are not prone to intense internal fracturing at temperatures as low as 250°C; this is because the release of H_2_O is gradual, only becoming critical at higher temperatures. The heat-induced failure in the matrix of the two Botswana samples must therefore be explained differently. In a comparative study between different types of chalcedony, Schmidt et al. [48] demonstrated that LS-chalcedony abruptly produces large quantities of H_2_O from 200°C onwards. The magnitude and promptness of this H_2_O release was found to be greater than in LF-chalcedony. This rapid release produces intense internal fracturing of the rock if heating rates are too fast or temperatures too high. In order to avoid thermal failure that would alter knapping quality, LS-chalcedony-rich rocks must be heat-treated extremely slowly and with great care, using relatively low temperatures. Thus, silcrete with a mixed LF- and LS-chalcedony cement, such as our Kalahari samples, must be heated with very different conditions than silcrete from the Cape (compare with [9]).

Our observations concerning pore space development upon heating within Kalahari silcrete samples are further supported by the results of our knapping experiment. While heat treatment of the South African silcrete produced smoother fracture surfaces up to a temperature somewhere between 400°C and 500°C, our Botswana sample showed the inverse trend. The increase in roughness of the fracture surfaces in the Botswana sample appears to result from the creation of internal fractures that offset the propagating fracture of a removal during knapping (for this mechanism see for example [51, 52]). We suspect that the presence of grain-supporting clasts (i.e. particles of Kalahari Sand; see Fig 3c and 3d), effectively acting as stabilising scaffolding, are the only reason why the silcrete from the Boteti River did not shatter into small unusable pieces during heating. Even without breaking down the whole rock, the high density of internal fracture surfaces attenuates propagating fractures and effectively reduces the knapping quality of the silcrete, leaving behind a disturbed and uneven fracture pattern.

In summary, our experimental results indicate that even heating to temperatures as low as 250°C generates fracturing within Kalahari samples. At higher temperatures fracturing becomes even more intense. This phenomenon significantly reduces the suitability of the silcrete as a tool-making stone. As such, heat treatment, whether achieved with relatively fast heating rates in an above-ground fire and temperatures close to 450°C [9–11, 13, 53], or even at slower heating rates in sediments beneath a fire at temperatures ranging from 350–500°C [3, 6], is unlikely to be advantageous for Kalahari silcrete. However, because systematic knapping experiments of both types of silcrete, before and after heat treatment, lie outside of the scope of this paper, our findings on knapping quality need to be corroborated by future systematic experimental studies.

## Conclusions and implications for the MSA of southern Africa

The results of this comparative study suggest that samples of silcrete from the Cape coastal zone and Kalahari respond fundamentally differently to heat treatment. While the former can be improved significantly as a tool-making stone by heat, the latter is deteriorated in terms of its suitability, to the extent that heat treating silcrete from the Kalahari would have been a counterproductive technology. We attribute this to differences in the petrography, crystallography and mode of genesis of silcrete in the two regions.

Our results may explain why there have been no previous suggestions of intentional heat treatment from MSA sites in Botswana or adjacent areas of the Kalahari in South Africa. There are reports of fire-damaged silcrete artefacts from the MSA layers of Rhino Cave [54] and White Paintings Shelter [55] in the Tsodilo Hills. These display characteristic indications of burning, such as crazing, incipient cracking, deterioration and colour change. However, it may be the case that the predominantly non-pedogenic silcretes of the Kalahari simply did not require heat treatment prior to use in tool manufacture. What is certain is that heat treatment would not have been beneficial for Kalahari silcrete.

On the basis of our findings, silcrete heat treatment should not be added as a new trait amongst the behaviours that characterise the MSA of the southern African subcontinent. For now, it remains unclear how far the silcrete heating techno-tradition would have extended beyond the Cape Coastal zone. It may, for example, have been a singularity confined to a small portion of the MSA cultural area in a relatively narrow zone of the western and southern Cape. Alternatively, it is possible that MSA peoples more widely knew of heat treatment but only applied it as a technical solution when necessary, according to the properties of specific silcrete sources. It also remains unclear how other varieties of non-pedogenic silcrete respond to heat treatment. While our Middle Kalahari drainage-line silcrete samples are representative of one of the dominant raw materials known to have been used by MSA peoples in the region, further experimental work is required on other non-pedogenic silcrete types (e.g. pan/lacustrine silcretes, such as those found around Lake Ngami; cf. [27]) before our results can be extrapolated.

Perhaps the most salient conclusion for archaeologists from this study is, to paraphrase George Orwell, that ‘all silcretes are not equal’. Finding that the same rock types were used in different archaeological contexts may suggest similarities in terms of raw material choice/preference or technical necessity. Our results, however, reveal the assumptions underlying this reasoning to be deeply problematic. While grouping Cape and Botswana samples under the term ‘silcrete’ is correct from a geological perspective, rocks from the two regions have very different properties and are unlikely to have been understood as being the same thing by MSA peoples. Instead, we echo Thiry and Milnes [56] in calling for a more nuanced consideration of silcrete characteristics, landscape associations, and origins in future technological studies.

## Supporting information

S1 Table Masses of the granulars obtained from each of the sample cubes, and thicknesses of the respective plan-parallel polished slabs used for transmission IR-spectroscopy. Values under ‘Total mass of the granular’ are the total mass of all fragments shattered from a cube. Values under ‘Mass of heat-treated Aliquot’ are the masses of the granular aliquots that were heat-treated to the temperatures indicated under ‘Cube label / heating temp.’. Values under ‘Mass of control Aliquot’ are the masses of the remaining aliquot that was not heated.
(XLSX)

S1 Fig The image acquisition rig used to capture images of silcrete samples. Note the custom sample holder in the foreground and the custom-built hemispherical light source for shadow-free, near-uniform illumination.
(TIF)

S2 Fig A typical captured image of a sample (labelled ‘01.450’) located within the custom sample holder. The sample is placed in its standardised location against the background formed by the X-Rite ColorChecker Passport Photo calibration target.
(TIF)

S3 Fig Linearity and normalisation verification after implementation of flat-field correction of scene illumination non-uniformity. (TIF)

S4 Fig Infrared spectrum of WK-13-13 between 5500 cm^-1^ and 2000 cm^-1^ compared to a kaolinite reference spectrum in the region of fundamental OH stretching vibrations. Note that the broad H2O absorption band of WK-13-13 is overgrown by two sharp OH stretching vibrations at 3695 cm-1 and 3620 cm-1 on its high frequency side. The bands correspond to the two OH groups of the 1:1 clay that is present as an impurity in this silcrete sample.
(TIF)

S5 Fig Williamsons-Hall diagrams of the four unheated samples. (TIF)

S1 Text Supporting Information for Colour analysis. (DOCX)
