# Supplementary figures and images for: Heat treatment as a universal technical solution for silcrete use? A comparison between silcrete from the Western Cape (South Africa) and the Kalahari (Botswana)
Source: PLoS One. 2017 Jul 19;12(7):e0181586. doi: 10.1371/journal.pone.0181586 (PMC5517054; doi:10.1371/journal.pone.0181586)

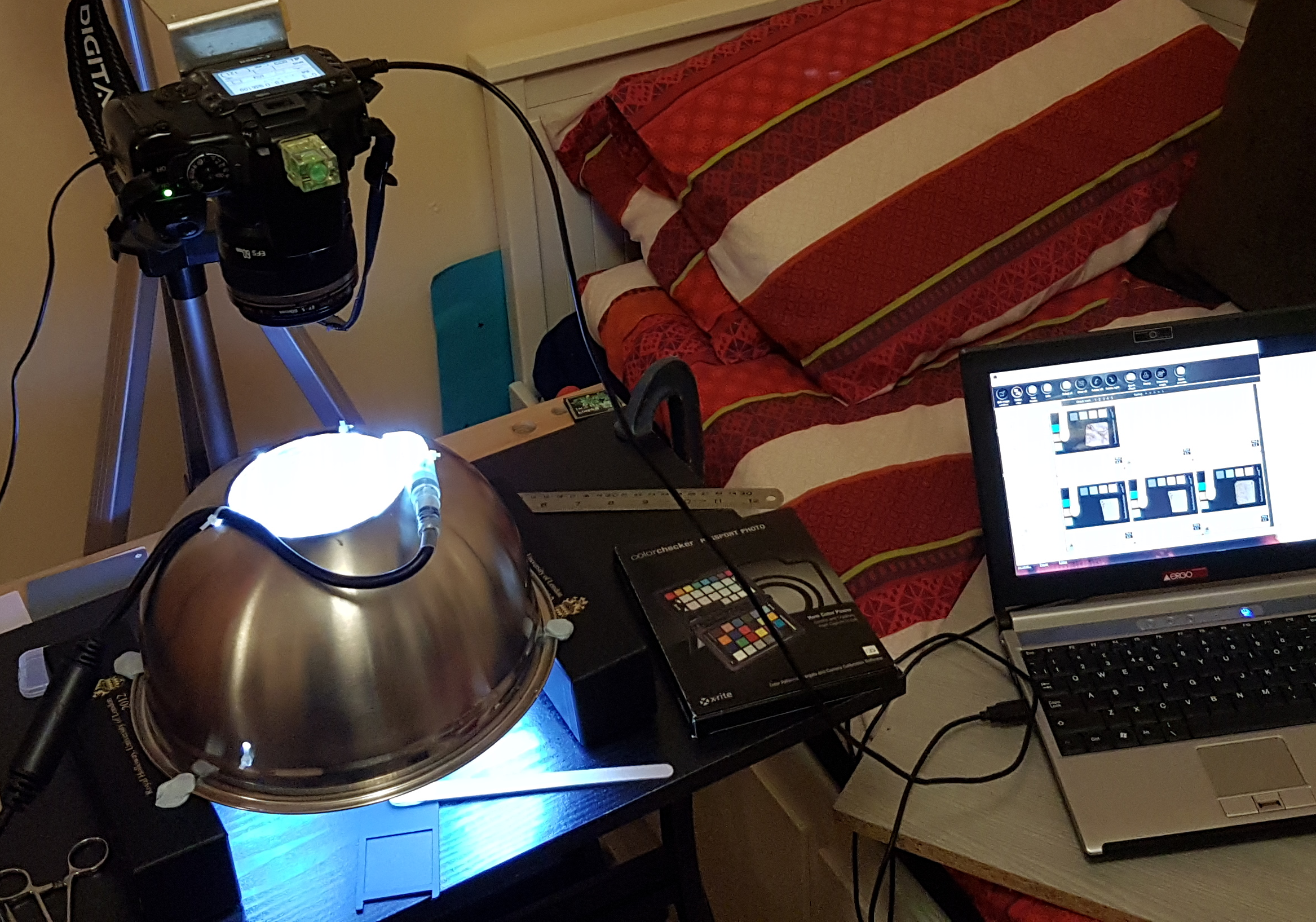

Supplement: S1 Fig — Note the custom sample holder in the foreground and the custom-built hemispherical light source for shadow-free, near-uniform illumination. (TIF) [file pone.0181586.s002.tif]

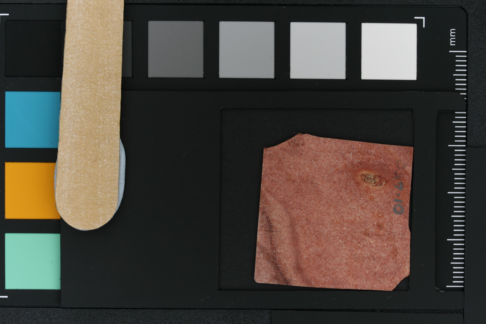

Supplement: S2 Fig — The sample is placed in its standardised location against the background formed by the X-Rite ColorChecker Passport Photo calibration target. (TIF) [file pone.0181586.s003.tif]

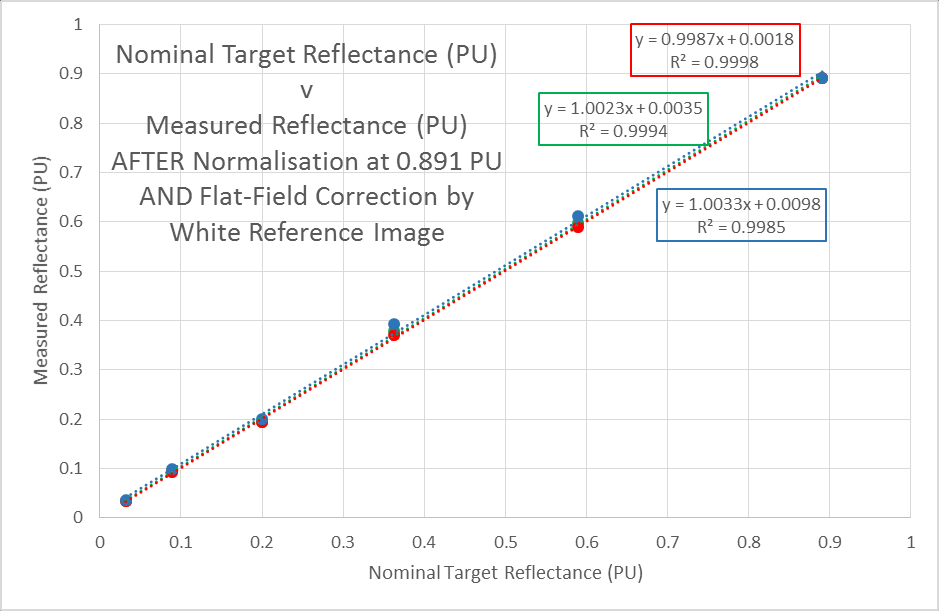

Supplement: S3 Fig — (TIF) [file pone.0181586.s004.tif]

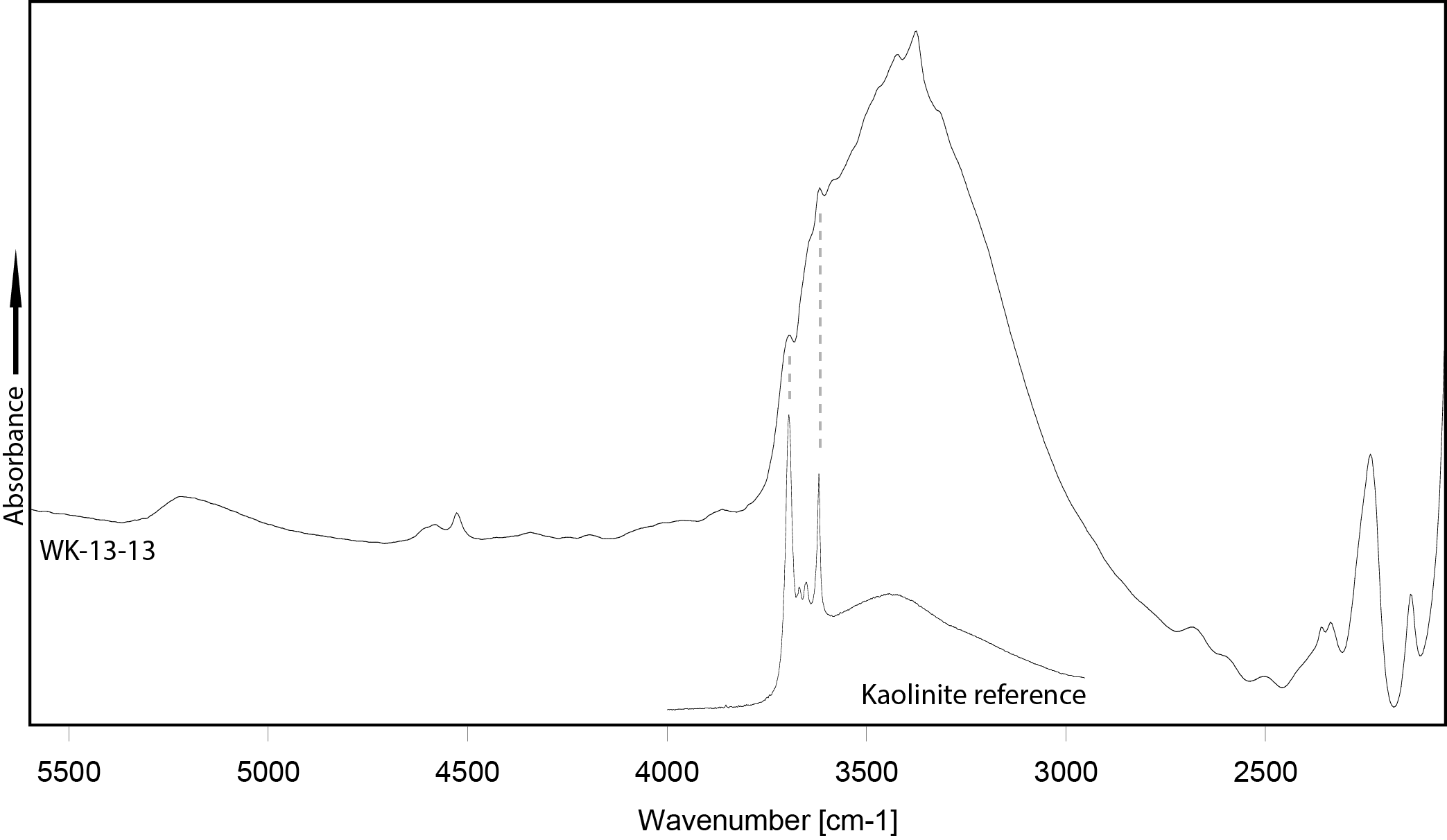

Supplement: S4 Fig — Note that the broad H2O absorption band of WK-13-13 is overgrown by two sharp OH stretching vibrations at 3695 cm-1 and 3620 cm-1 on its high frequency side. The bands correspond to the two OH groups of the 1:1 clay that is present as an impurity in this silcrete sample. (TIF) [file pone.0181586.s005.tif]

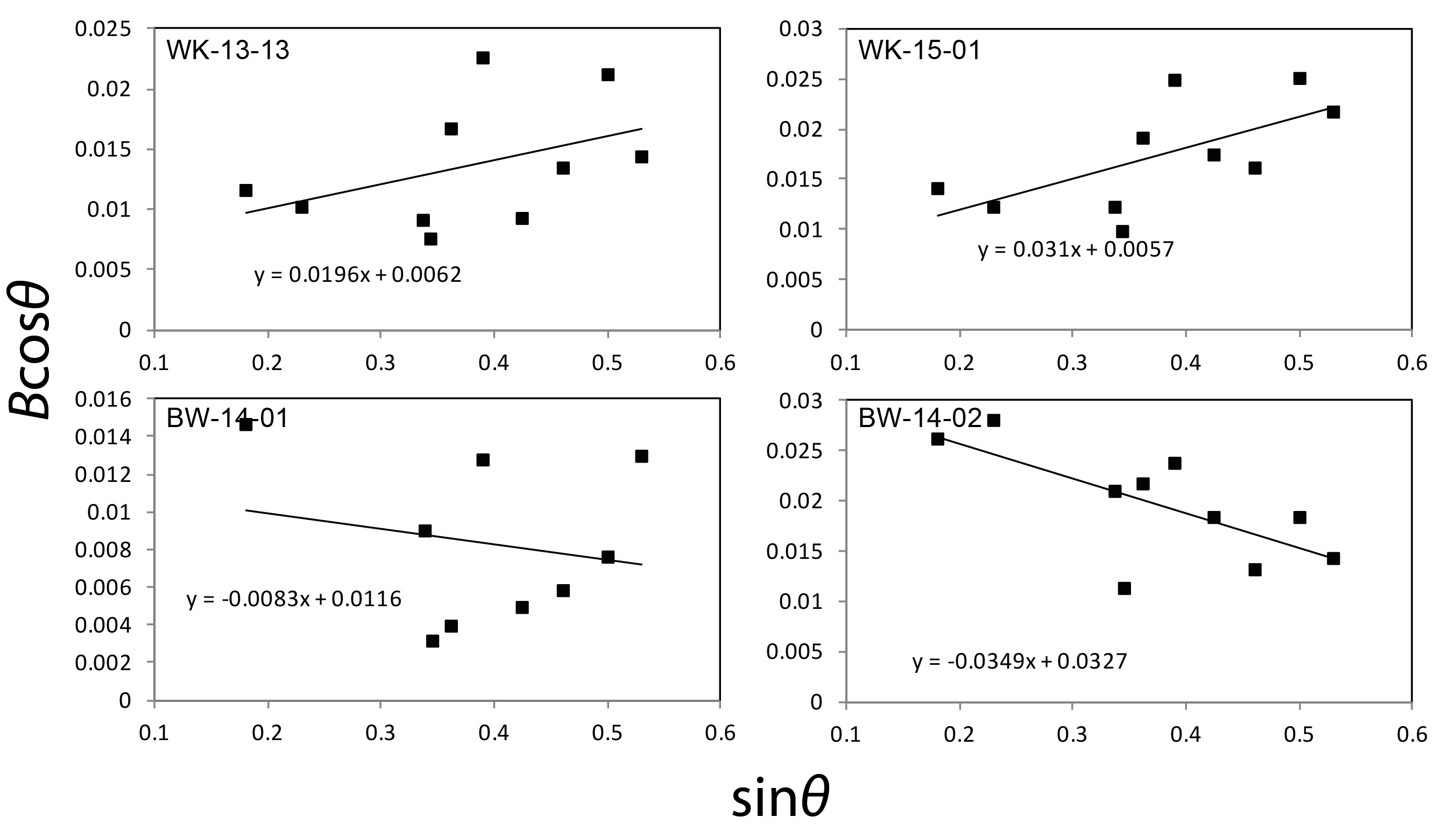

Supplement: S5 Fig — (TIF) [file pone.0181586.s006.tif]
