# Supplementary material for: Heat treatment as a universal technical solution for silcrete use? A comparison between silcrete from the Western Cape (South Africa) and the Kalahari (Botswana)
Source: PLoS One. 2017 Jul 19;12(7):e0181586. doi: 10.1371/journal.pone.0181586 (PMC5517054; doi:10.1371/journal.pone.0181586)
Supplement: S1 Text — (DOCX) [file pone.0181586.s007.docx]

**S1 Text.**

**Supporting Information for Colour Analysis**

Patrick Schmidt ^a, b^, David J. Nash ^c, d^ Sheila Coulson ^e^, Matthias Göden ^a^, Graeme J. Awcock ^c^

^a^ Eberhard Karls University of Tübingen, Department of Prehistory and Quaternary Ecology, Schloss Hohentübingen, 72070 Tübingen, Germany.

^b^ Eberhard Karls University of Tübingen, Department of Geosciences, Applied Mineralogy, Wilhelmstraße 56, 72074 Tübingen, Germany.

^c^ School of Environment and Technology, University of Brighton, Lewes Road, Brighton BN2 4GJ, United Kingdom.

^d^ School of Geography, Archaeology and Environmental Studies, University of the Witwatersrand, Private Bag 3, Wits 2050, South Africa.

^e^ Department of Archaeology, Institute of Archaeology, Conservation, and History, Blindernveien 11 (Post Box 1019) University of Oslo, 0315 Oslo, Norway.

**Silcrete image acquisition and processing strategy**

The image acquisition process adopted the ‘Scene Constraints’ principles described in Awcock and Thomas (1995) to eliminate variability and to standardise image acquisition; with each element selected to better support calibration, as follows:

**Physical constraints**

A custom image acquisition rig was constructed (**S1 Fig**), with fixed datums established on the base-board, and used with a custom-built sample holder, to ensure that samples were reliably located in repeatable location in the camera field-of-view (FOV) in relation to a background of suitable calibration elements (see below). A near hemispherical light source was constructed to deliver a best approximation of shadow-free, near-uniform illumination. ‘White’ LEDs were utilised in the design to stimulate all colours in scene.

**Camera/Sensor**

A Canon 400D SLR with 10.1 Mpixel sensor was used to capture the images. This model is specifically featured as a suitable camera that has been characterised in Troscianko and Stevens (2015). It is capable of saving RAW and JPEG images, simultaneously, onto its internal memory card. A Canon EF-S 60 mm focal length specialist ‘Macro’ lens was fitted to the camera. This is a top-quality lens for distortion-free close-up work. The combination of lens and lighting ensure that there is no opportunity for lens flare (referred to as veiling glare in Troscianko and Stevens, 2015).

The camera was custom white-balanced, using a neutral target from the ColorChecker to ensure that the JPEG images had no colour bias. RAW images were collected for the critical image analysis work, since they can be processed by a RAW convertor to deliver 16-bit linear outputs. As per Troscianko and Stevens’ toolbox (2015), ij-dcraw (Sacha, 2013), a plugin for ImageJ that uses dcraw (Coffin, 2015), was utilised to extract pixel data from RAW files in a linear fashion (Chakrabarti et al, 2009).

To eliminate the possibility of camera shake, or of disturbing the fixed FOV, the camera was controlled remotely using the Canon EOS utility (Canon, 2017) to trigger the shutter. This also gained the advantage that images were backed-up on the computer’s hard disc, as well as on the camera’s internal memory card.

**Calibration target**

An X-Rite ‘ColorChecker passport photo’ (Part No MSCCPP, Serial No. 222667; X-Rite, 2017) calibration target was fixed to the base-board and used as a background for the images. Its location in the camera FOV was chosen such that five of its six neutral greyscale targets could be acquired simultaneously with the sample-under-test, along with a millimetre scale for calibration purposes (**S2 Fig**).

The X-Rite ColorChecker passport photo is the latest evolution of the Gretag-Macbeth calibration target, which is traceable to the National Institute of Standards and Technology (NIST) through the manufacturer’s laboratory competencies and processes that comply with ISO 17025. It is designed for field use. It provides colour-neutral greyscale calibration patches of known and warranted reflectance, of 0.891, 0.589, 0.363, 0.200, 0.089 and 0.032 per-unit reflectance, respectively.

**White reference target for Flat Field Correction**

Since the calibrated grey-scale patches provided by the X-Rite target are placed at different locations within the FOV, it is necessary for proper calibration and normalisation that the whole FOV is illuminated uniformly. However, despite the use of a near-hemispherical light source, non-uniformities in lighting are inevitable; these must be corrected by using a suitable form of ‘Flat-Field-Correction (FFC)’ of images (Alper, 2011), post-capture. In order to correct lighting non-uniformities, a low-(spatial) frequency form of FFC is required (Koene, 2016a; Koene, 2016b), whereby the image to be corrected is divided by the image of a uniform white target before normalisation.

A piece of good-quality, uniformly white, card was placed in the image plane such that it filled the FOV, and an image was captured for use in a ‘Flat-Field-Correction (FFC)’ algorithm that was implemented as part of the normalisation strategy. By incorporating this FFC step into the normalisation process before the images were normalised to the 0.891 PU grey-scale patch that appears within each image, it was not necessary to ensure that the white reference target has a neutral colour balance.

**Exposure**

Given the largely 2-dimensional nature of the scene, large depth-of-field (DOF) was not a priority and thus any aperture could be selected; f8 was chosen because this typically gives optimum lens performance (best DOF, with minimal diffraction artefacts). Given the constrained scene and remote triggering of the shutter, a relatively long 1/3 s exposure could be selected to bring the exposure to a value that would just prevent saturation of the sensor (i.e. give good scene brightness, without over-exposing the samples, or more likely, the calibration targets).

**Post-capture image processing**

After capture, the JPEG and RAW images were subjected to different processing workflows, each relevant to their intended purpose. The JPEG images were used to develop an array of 36 thumbnail images to show the whole set of heat-treated samples (**Fig 2** of the main text). The JPEGs were white-balanced, in-camera, using a custom white balance setting that was set using the X-Rite ‘ColorChecker Passport Photo’ large-area colour-neutral white reference. The array also incorporates a subset of the greyscale reference patches from the colorchecker target, on a per-batch basis, to provide reassurance of white-balance and exposure throughout any subsequent reproduction pathway.

During their creation, JPEG images are subjected to a non-linear light-to-output-brightness mapping that is intended to suit perception by the human visual system (HVS). This makes the JPEG images inherently unsuitable for quantitative analysis of colour shift, as does the ‘lossy’ compression algorithm that is applied each time a JPEG image is saved. By contrast, RAW images can be processed to produce 16-bit outputs, where the output value is linearly related to scene brightness, by using a RAW image convertor. This takes the raw data from the camera sensor and de-mosiacs it to produce 16-bit linear images for each of the Red, Green and Blue (R,G,B) colour channels, respectively. Troscianko and Stevens (2015) have demonstrated how such images can be processed and exploited for ecological purposes with the aid of the ‘DCRAW’ plugin (which delivers 16-bit linear images from the RAW files) and a ‘toolbox’ that they have created within the ImageJ image processing environment. Troscianko and Stevens (2015) have additionally created other specialised plugins to normalise the images to absolute reflectance values with the aid of calibrated targets included within the FOV of the images. This toolbox provided a very useful starting point for the development of custom processing workflows for the silcrete sample images. However, there was no opportunity built into the Troscianko and Stevens processing strategies to compensate for non-uniformity in scene illumination that is all-but impossible to avoid, and which would have a harmful effect upon the linear relationship between scene reflectance and recorded pixel level.

The coding of Troscianko and Stevens plugins in ImageJ’s scripting language (Mutterer and Rasband, 2012) meant that they could be ‘reverse-engineered’ to identify the key elements of the workflow that were required for normalisation. New custom scripts were developed that integrated compensation for lighting non-uniformity into the workflow. This was achieved with the aid of (low spatial frequency) ‘Flat-Field-correction (FFC)’ strategy, with the support of a ‘white-reference’ image. As described above, this image is taken, using identical scene constraints to those applied to the sample images, of a uniform brightness target that fills the field of view of the camera. This is a way of encoding the spatial non-uniformity of the scene lighting. The impact of spatial non-uniformity of the illumination is then minimised by dividing each sample image, on a pixel-by-pixel, spatially-correlated, basis by the white reference image. If FFC is implemented before normalisation of the sample image to a neutral grey reference patch of known reflectance, then the white balance of the white-reference target is non-critical. As part of the process validation, linearity of the processed output images was checked using a calibration reference image, which contained six neutral grey target patches of known reflectance; viz 0.891, 0.589, 0.363, 0.200, 0.089 and 0.032 per-unit reflectance, respectively, from the X-Rite ‘ColorChecker Passport Photo’. This confirmed that the linearity of the processed outputs was improved by the use of FFC, with r^2^ values of the linear fit raised to 0.9998, 0.9994 and 0.9985 for the R, G and B channels, respectively (**S3 Fig**).

RAW images of all silcrete 36 samples were processed in this way by the custom script, and were then subjected to investigation of red-dominance. Wherever possible, a common 396 x 405 region-of-interest (ROI) was used as the target of analysis. There were some notable exceptions to this, where the sample slice was unusually small or irregular in shape, and in one case, where the sample appeared to include weathered host rock for more than half of the sample slice (i.e. the slice from sample WK-13-13 that was heated to 350°C; see **Fig 2** of the main text).

A semi-automated scripted process was used to perform cropping and rotation of all the normalised sample images. For each sample, the script paused to allow the user to manually scrutinise the Red, Green and Blue histograms of the analysis ROI for selection of the dominant modal value for each colour; this was collected into a spreadsheet. The dominant mode was selected as a strategy to eliminate bias in the mean value caused by non-silcrete inclusions that were prevalent in some images, and which even led to evidence of multi-modality in some histograms. The collection of R, G and B modes represented an effective data-reduction strategy that identifies a point in R,G,B space to objectively characterise the colour of the sample. This characteristic colour could then be analysed for evidence of red-dominance, and of shift of red-dominance in a way that is linked to the treatment temperature of the silcrete samples.

**Colour quantification**

Since the absolute R, G, B modal values are significantly influenced by reflectance variations of the samples, and our interest is in colour dominance and shift, it was not appropriate to simply plot the characteristic R,G,B values against sample treatment temperature. Instead, methods were sought to eliminate the influence of scene intensity. Various strategies were explored based on ‘saturating’ the characteristic R,G,B values by subtracting the min{R,G,B} from each value. In all samples the min{R,G,B} was always the Blue value, confirming that the colour of all samples was dominated by red, and thus characterised entirely in the R-G colour plane. As a result, the saturated Red reflectance was plotted against sample treatment temperature, in batches, for various derived parameters; viz ‘magnitude of Saturated Red as % of sum of Saturated Red + Saturated Green’, ‘magnitude of Saturated Red as % of magnitude of Saturated Red / Saturated Green vector’ and ‘Phase angle of the Saturated Red / Saturated Green vector’. All of these metrics yielded similar results, which suggest that reddening increases with sample treatment temperature, then stabilises, before ultimately going into decline.

The most common way to analyse colour in a way that is independent of brightness and saturation is to transform the reflectance information from R,G,B space into HSI (Hue, Saturation, Intensity) space. In this space, Hue encodes all information about what humans loosely call ‘colour’, and quantifies it in a circular form, where 0 degrees indicates that pure red is the dominant wavelength. This transformation was accomplished using the equations specified by Gonzalez and Woods (2008, section 6.2.3), and the Hue was plotted against sample treatment temperature. Once again, the curves showed the same tendency as the other metrics, albeit in an inverse relationship whereby the Hue angle reducing towards 0 degrees indicates a shift towards red-dominance.

Ultimately, we decided that plotting Hue against temperature might prove confusing, so we elected to plot the magnitude of Saturated Red as percentage of magnitude of Saturated Red / Saturated Green vector in **Fig 4** (of the main text) as the final representation of the shift of colour towards red against temperature.

**S1 Text References:**

Alper, G. (2011) Flat field correction improves machine vision camera uniformity, <http://info.adimec.com/blogposts/bid/43199/Flat-field-correction-improves-machine-vision-camera-uniformity>; - last accessed 19/5/2017.

Awcock, G. J. and Thomas, R. (1995). *Applied Image Processing*. Houndmills, Basingstoke, UK: Macmillan Press Ltd.

Canon (2017). Canon EOS Utility 2 <http://www.canon.co.uk/support/consumer_products/products/cameras/digital_slr/eos_400d.aspx?type=software&softwaredescriptionid=tcm:14-1330031&os=WINDOWS%2010%20(64-bit)&language> ; - last accessed 19/5/2017.

Chakrabarti, A., Scharstein, D. and Zickler, T. (2009) An empirical camera model for internet color vision. *Proc. BMVC*, pp. 51.1–51.11.

Coffin, D. (2015). DCRAW V. 9.26 [https://www.cybercom.net/~dcoffin/dcraw/](https://www.cybercom.net/%7Edcoffin/dcraw/); - last accessed 19/5/2017.

Gonzalez, R. C. and Woods, R. E. (2008). *Digital Image Processing*. 3^rd^ Edn. Upper Saddle River, New Jersey, USA: Pearson Prentice Hall.

Koene, B. (2016a). Which types of flat field corrections exist and why it matters for high resolution cameras? <http://info.adimec.com/blogposts/which-types-of-flat-field-corrections-exist-and-why-it-matters-for-high-resolution-cameras>; - last accessed 19/5/2017.

Koene, B. (2016b). How to use Flat Field Correction in practice? <http://info.adimec.com/blogposts/how-to-use-flat-field-correction-in-practice>; - last accessed 19/5/2017.

Mutterer, J and Rasband, W (2012), *ImageJ Macro Language Programmer’s Reference Guide v1.46d***,** available online for download from <http://rsbweb.nih.gov/ij/docs/macro_reference_guide.pdf>; - last accessed 22/1/2017.

Sacha, J. (2013) ij-dcraw plugin, V. 1.4.0, <http://sourceforge.net/projects/ij-plugins/files/ij-dcraw/>; - last accessed 19/5/2017.

Troscianko, J. and Stevens, M. (2015). Image calibration and analysis toolbox – a free software suite for objectively measuring reflectance, colour and pattern. *Methods in Ecology and Evolution*. 6. 1320-1331.

X-Rite (2017). X-Rite ColorChecker Passport Photo <http://www.xrite.com/categories/calibration-profiling/colorchecker-passport-photo>; - last accessed 19/5/2017.
